# Proteomic Characterization of Replication Stress and Impaired Antioxidant Defense in Tacrolimus-Induced Chronic Nephrotoxicity

**DOI:** 10.3390/ijms27157030

**Published:** 2026-08-05

**Authors:** Tamaki Ishima, Sho Nishida, Shota Tomida, Risa Watanabe, Daiki Iwami, Kenichi Aizawa

**Affiliations:** 1 Department of Translational Research, Clinical Research Center, Jichi Medical University Hospital, Shimotsuke 329-0498, Japan; 2 Division of Renal Surgery and Transplantation, Department of Urology, Jichi Medical University, Shimotsuke 329-0498, Japan; 3 Clinical Pharmacology Center, Jichi Medical University Hospital, Shimotsuke 329-0498, Japan

**Keywords:** tacrolimus nephrotoxicity, proteomics, antioxidant defense, replication stress, mitochondrial dysfunction, lipid metabolism

## Abstract

Tacrolimus (TAC) nephropathy is a major complication of immunosuppressive therapy and contributes to chronic kidney disease (CKD) progression through ischemia, metabolic dysfunction, and oxidative stress; however, its protein-level basis remains unclear. This study sought to identify characteristic molecular alterations in renal cortices of TAC-treated mice, so as to clarify the link between replication stress responses and metabolic dysfunction. A previously generated proteomic dataset from a TAC-induced chronic nephrotoxicity mouse model was analyzed using a protein-centered analytical strategy, including statistical, Gene Ontology, pathway, upstream regulator, and disease-enrichment analyses. A total of 7466 proteins were quantified. Upregulated proteins included KAT6A and NCKAP1, whereas downregulated proteins included NDUFC2, HSD17B12, and TECR. Coordinated impairment of CoQ_10_-dependent and glutathione-dependent antioxidant defenses was identified, reflected by reductions in AIFM2 (FSP1) and GSTA4/GSTT2. Enrichment analyses indicated activation of MCM- and ATR-associated replication stress responses in the upregulated group, and impaired lipid metabolism, CoA biosynthesis, mitochondrial function, and redox regulation in the downregulated group. TAC nephropathy is characterized by two major molecular signatures: central disruption of antioxidant defense systems, spanning FSP1-mediated CoQ_10_ regeneration and GST- associated antioxidant systems, together with suppression of lipid and energy metabolism and activation of replication stress responses. These findings provide a protein-level molecular framework linking coordinated impairment of antioxidant defense systems, suppression of lipid and energy metabolism, and activation of replication stress responses in TAC-induced chronic nephrotoxicity. These findings also suggest the FSP1 pathway, GST-associated antioxidant systems, and CoA-dependent metabolism as potential therapeutic targets for CKD progression.

## 1. Introduction

Tacrolimus (TAC) [1] is a cornerstone immunosuppressant in solid organ transplantation, including kidney transplantation, and has substantially improved short-term graft survival by preventing acute rejection. However, TAC-induced chronic nephrotoxicity remains a major limitation to long-term graft survival [2,3,4]. TAC-induced chronic nephrotoxicity is characterized by arteriolar hyalinosis and tubulointerstitial fibrosis, leading to irreversible chronic kidney injury [5,6]. Despite its clinical importance, the molecular basis of TAC-induced chronic nephrotoxicity, particularly at the level of metabolic regulation and antioxidant defense, remains incompletely defined.

To characterize the pathophysiology of TAC-induced chronic nephrotoxicity, we previously conducted metabolomic analyses of renal tissue and blood [7,8], lipidomic profiling [9], and an integrated trans-omic analysis [10]. Although transcriptomic–proteomic integration helped to infer upstream transcriptional regulators, proteomic information was used largely in a secondary role. Consequently, the biological significance of individual protein alterations and their coordinated functional changes were not systematically investigated. Moreover, pronounced discrepancies between mRNA and protein levels were observed: 33 transcripts were upregulated while their proteins were downregulated, and 26 transcripts were downregulated while their proteins were upregulated. These mismatches are consistent with reports showing weak mRNA–protein correlation in kidney disease [11]. Gene Ontology (GO) enrichment also yielded discordant interpretations across layers, suggesting that transcriptome-centered analyses alone cannot capture functional consequences of TAC exposure. These observations highlighted how little we understand about TAC nephrotoxicity: despite comprehensive trans-omic profiling, it remained unclear whether coordinated protein-level alterations can reveal pathogenic mechanisms that are not apparent from transcriptome-centered analyses. To address this matter, the present study was designed as a protein-centered investigation using a previously generated proteomic dataset. By systematically evaluating statistically significant protein alterations and their biological implications, this complementary analytical strategy extends our previous metabolomic, lipidomic, and trans-omic investigations.

Importantly, TAC-induced chronic nephrotoxicity involves multilayered cellular stress responses, including replication stress, mitochondrial and lipid metabolic dysfunction, and impaired antioxidant defenses. However, whether these pathological processes converge upon a common molecular framework at the protein level remains unclear. How these processes are altered at the protein level, where redox control, membrane remodeling, and metabolic fluxes are directly regulated, has not been systematically investigated. A comprehensive protein-level understanding is essential, given that key determinants of oxidative vulnerability, such as coenzyme Q (CoQ)_10_ availability, glutathione S-transferase (GST) activity, mitochondrial enzyme integrity, and lipid metabolic enzymes, cannot be inferred solely from transcript levels.

Accordingly, we performed a comprehensive protein-centered investigation using a previously generated renal cortical proteomic dataset to delineate the protein-level molecular architecture of TAC-induced chronic nephrotoxicity. Our analyses identified coordinated protein-level alterations underlying replication stress, mitochondrial dysfunction, impaired lipid metabolism, and disruption of antioxidant defense systems in TAC-induced chronic nephrotoxicity. Furthermore, we identified coordinated breakdown of metabolic and membrane-associated antioxidant networks, including the FSP1–CoQ10 and GST pathways, thereby providing complementary mechanistic insights into TAC-induced chronic nephrotoxicity beyond those obtained from our previous trans-omic analysis.

## 2. Results

### 2.1. Analysis Using Perseus

#### 2.1.1. Filtering Based on Fold-Change Thresholds

After log_2_ transformation and imputation of missing values, a two-group *t*-test was performed. Proteins with *p* < 0.05 and a log_2_ fold change ≥ 1 were classified as upregulated proteins (Cluster No. 1), whereas those with *p* < 0.05 and a log_2_ fold change ≤ −1 were classified as downregulated proteins (Cluster No. 2). Under these criteria, 27 proteins were classified as upregulated and 64 proteins as downregulated (Figure S1).

#### 2.1.2. Filtering Based on FDR Thresholds

After log_2_ transformation and imputation of missing values, a two-group *t*-test was performed with a significance threshold of *p* < 0.05. To correct for multiple comparisons, a permutation-based false discovery rate (FDR) approach (250 permutations; FDR 0.05; S_0_ = 0.1) was applied. A volcano plot was generated based on these results, and proteins with FDR < 0.05 were highlighted. Two distinct clusters with opposite regulation patterns were identified. Under these criteria, 34 proteins were upregulated, and 90 were downregulated (Figure S2).

**Figure 1:**
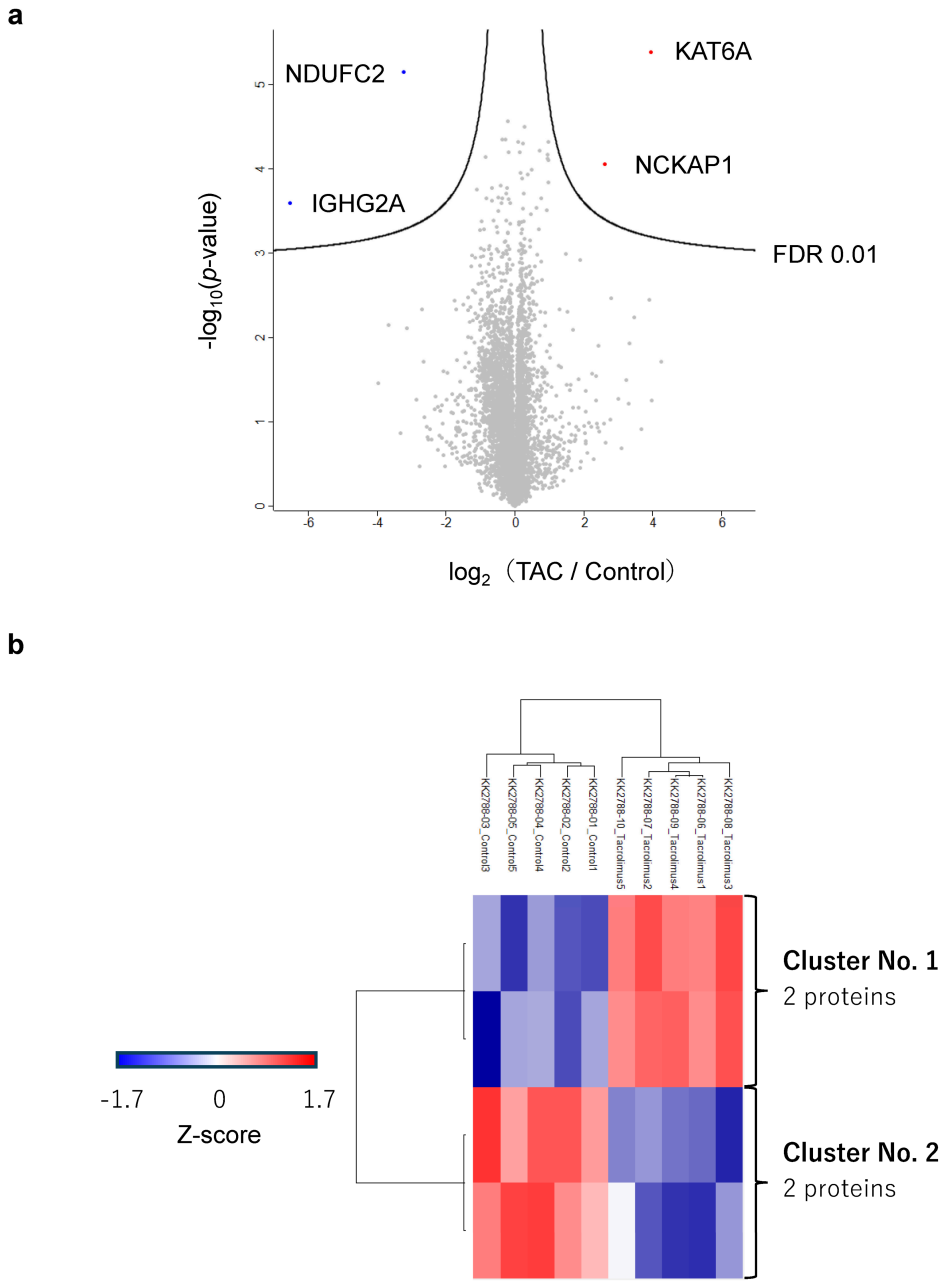
Differentially expressed proteins identified with permutation-based FDR < 0.01. After log_2_ transformation and missing-value imputation, a *t*-test was performed. Multiple testing correction was conducted using permutation-based FDR (250 permutations; FDR threshold = 0.01; S_0_ = 0.1). (**a**) Volcano plot of protein expression changes. The *y*-axis shows –log_10_ (*p*-values), and the *x*-axis shows log_2_ (TAC/Control). The line indicates the FDR = 0.01 threshold. Red dots represent upregulated proteins, blue dots represent downregulated proteins, and grey dots indicate non-significant proteins. (**b**) Proteins with FDR < 0.01 were selected, and their quantitative values were converted to Z-scores to generate the heatmap.

When a more stringent threshold of FDR < 0.01 was applied, results were further restricted to four proteins: Histone acetyltransferase KAT6A (KAT6A) and Nck-associated protein 1-like (NCKAP1) were upregulated, and Ig gamma-2A chain C region, A allele (IGHG2A), and NADH:ubiquinone oxidoreductase subunit C2 (NDUFC2) were downregulated (Figure 1). These four proteins represent the most statistically robust TAC-induced alterations, as they remained significant even at FDR < 0.01.

The FDR < 0.01 protein set was used as a stringent sensitivity analysis to evaluate the robustness of the findings identified in the primary FDR < 0.05 dataset.

### 2.2. Bioinformatic Analyses of Proteins Upregulated by TAC

In total, 7466 proteins were detected. Among these, 34 were classified as upregulated, based on permutation-based FDR < 0.05 criteria. These proteins were subjected to analyses described below.

#### 2.2.1. GO Enrichment Analysis

GO term enrichment analysis was performed to identify characteristic functional annotations associated with proteins that showed differential expression. Enrichment analyses were conducted for GO categories, *Biological Process*, *Cellular Component*, and *Molecular Function*.

**Figure 2:**
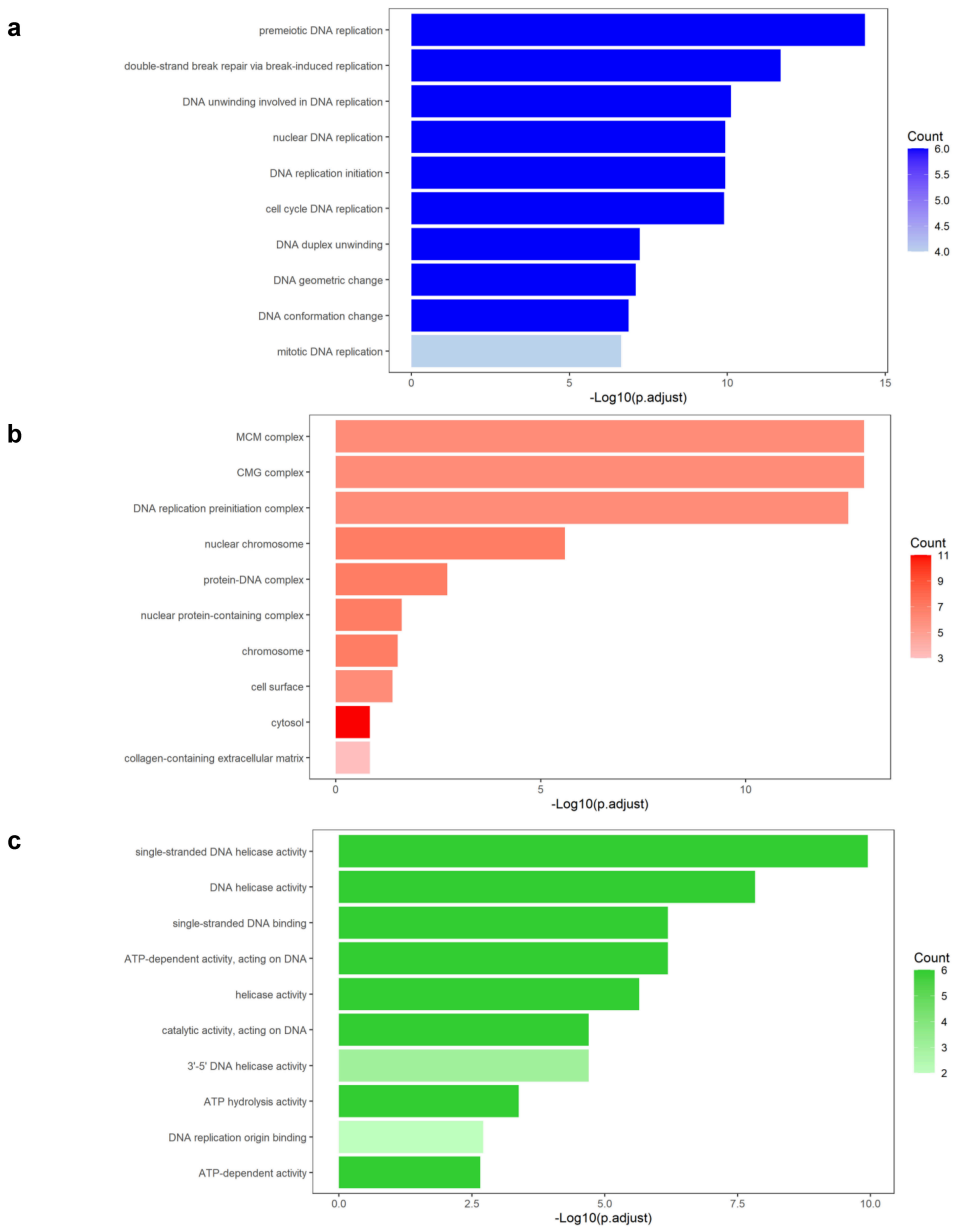
GO enrichment analysis of proteins upregulated in the TAC-treated group. Bar graphs show the 10 enriched GO terms most strongly associated with upregulated proteins in each GO category, based on adjusted *p*-values from enrichment analysis: (**a**) *Biological Process*, (**b**) *Cellular Component*, and (**c**) *Molecular Function*.

The 10 GO terms with the highest relevance to altered proteins, based on database-matched enrichment results, were visualized using bar charts (Figure 2). In addition, tree maps were generated to provide an overview of more granular GO terms (Figures S3–S5).

##### Biological Process

The top 10 enriched GO terms predominantly comprised subcategories related to intermediate-level terms: *DNA unwinding involved in DNA replication* (GO:0006268), *premeiotic DNA replication* (GO:0006279), and *double-strand break repair via break-induced replication* (GO:0000727).

Another highly enriched intermediate category was *detoxification of copper ion* (GO:0010273), which reflected enrichment of Mt1 and Mt2 (Figure S3).

##### Cellular Component

The top 10 enriched GO terms were largely comprised of subcategories *mini-chromosome maintenance* (MCM) *complex* (GO:0042555), *CMG complex* (GO:0071162), *protein–DNA complex* (GO:0032993), *external side of plasma membrane* (GO:0009897), *nuclear protein-containing complex* (GO:0140513), *collagen-containing extracellular matrix* (GO:0062023), and *cytosol* (GO:0005829) (Figure S4).

##### Molecular Function

The top 10 enriched molecular functions consisted mainly of subcategories *single-stranded DNA helicase activity* (GO:0017116), *ATP hydrolysis activity* (GO:0016887), *single-stranded DNA binding* (GO:0003697), and *DNA replication origin binding* (GO:0003688) (Figure S5).

#### 2.2.2. Pathway Enrichment Analysis

**Figure 3:**
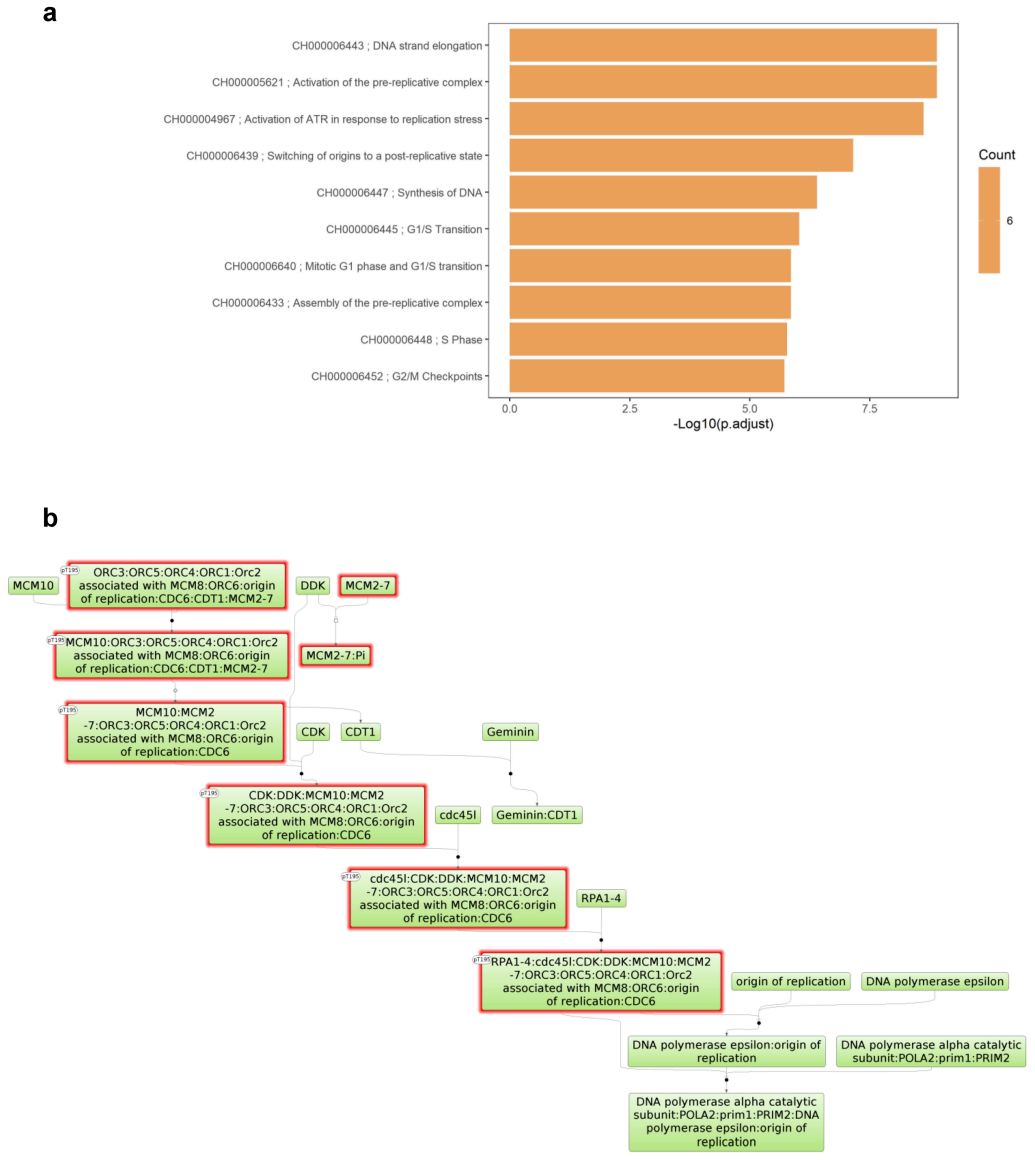
Pathway analysis of proteins upregulated in the TAC-treated group. (**a**) The top 10 enriched pathways identified by enrichment analysis are shown as a horizontal bar graph. Bars represent the enrichment score, and pathway names are listed on the *y*-axis. (**b**) A representative pathway diagram illustrating key molecular interactions and components involved in TAC-responsive processes, with particular emphasis on *Activation of the pre-replicative complex pathway*. This pathway was among the most highly enriched categories identified in this analysis. In the diagram, green, rounded rectangles represent macromolecules, indicating either individual proteins or protein complexes and related structures. Those outlined in red correspond to molecules significantly associated, based on the enrichment analysis. A small capsule-shaped element positioned at the upper left of a green, rounded rectangle, labeled “pT195,” denotes phosphorylation at threonine residue 195. Regarding edges, a black arrow indicates production, an arrowhead with a white circle represents catalysis, a black circle on the edge denotes an association reaction, a double circle indicates a dissociation reaction, and a white-outlined square on the edge represents a process.

Pathway enrichment analysis was performed to characterize differences at the pathway level by mapping upregulated proteins to known metabolic, signaling, membrane transport, and other curated pathways in the database. Enrichment analysis was conducted against all registered pathways, and the top 10 enriched pathways were visualized using bar charts. In addition, the three most statistically significant pathways, *Activation of the pre-replicative complex pathway*, *DNA strand elongation*, and *Activation of Ataxia Telangiectasia and Rad3-related (ATR) in response to replication stress*, were also illustrated (Figure 3, Figures S6 and S7).

#### 2.2.3. Upstream Enrichment Analysis

**Figure 4:**
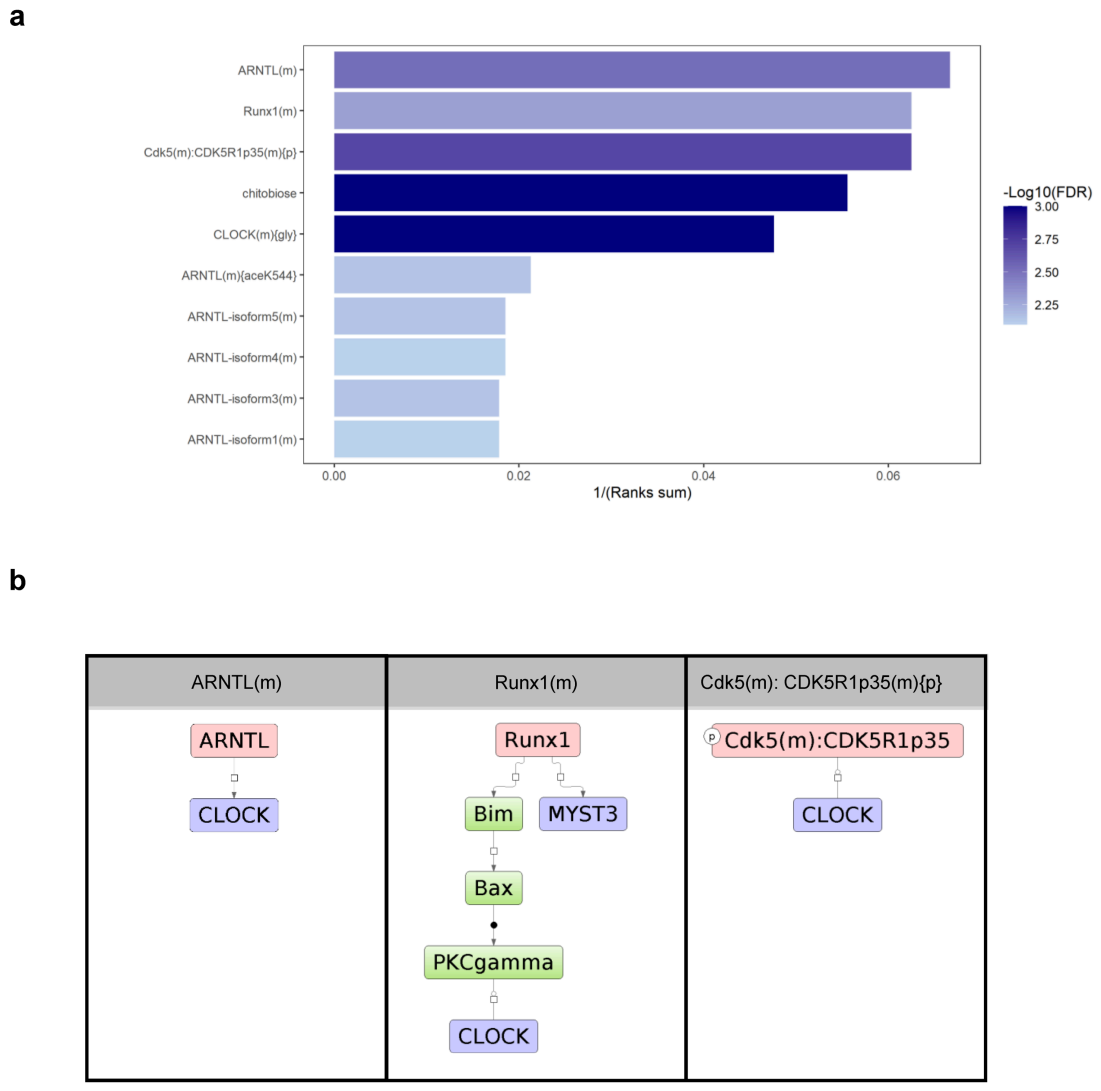
Upstream regulator analysis of proteins upregulated in the TAC-treated group. (**a**) The top 10 regulators identified by upstream analysis are shown as a horizontal bar graph. Bars represent the inverse of the ranking score [1/(Ranks sum)], and master molecule names are displayed on the *y*-axis. (m) indicates motif-based predictions derived from sequence motif analysis. {p} denotes phosphorylated molecules, suggesting that they function in a phosphorylated state. {gly} indicates glycosylated molecules, meaning that they function in a glycosylated state. {aceK544} refers to acetylation at lysine 544. (**b**) Representative diagrams for the top three key regulators are shown. Rounded rectangles indicate molecule types: pink for master regulatory molecules, purple for input molecules used in network analysis, and green for intermediate molecules predicted by the algorithm. A small circle containing the letter “p” in the upper-left corner of a rounded rectangle denotes phosphorylation. For edges, black arrows indicate production; white squares along arrows represent processes; white circles at arrowheads indicate catalysis; and black circles along arrows represent associations.

Upstream enrichment analysis identified the 10 upstream regulatory factors most likely responsible for upregulated proteins, and results were visualized as a bar chart. This analysis detected several key regulators, including circadian clock–related factors ARNTL (multiple isoforms), CLOCK, the transcription factor RUNX, and the kinase Cdk5/CDK5R1 (p35). These molecules exhibited various post-translational modifications, such as acetylation (ARNTL K544), glycosylation (CLOCK), and phosphorylation (CDK5R1). In addition, chitobiose, a glycan-related factor, was also identified. Upstream regulatory networks for the top three candidate factors are illustrated in Figure 4.

#### 2.2.4. Disease Enrichment Analysis

Although this analysis was performed using kidney tissue from TAC-treated mice, disease enrichment was conducted using a human disease–associated protein database to identify conditions linked to upregulated proteins. The most enriched terms included several tumor-related categories such as *Medulloblastoma*, *Mesothelioma*, and *Hodgkin disease* (Figure S8). Representative proteins upregulated upon TAC treatment that contributed to these disease terms included MCM2–7, AKT3, NES, and VCAM1.

### 2.3. Bioinformatic Analyses of Proteins Downregulated by TAC

Among 7466 proteins detected, 90 were defined as downregulated, based on permutation-based FDR < 0.05. These proteins were subjected to analyses described below.

#### 2.3.1. GO Enrichment Analysis

**Figure 5:**
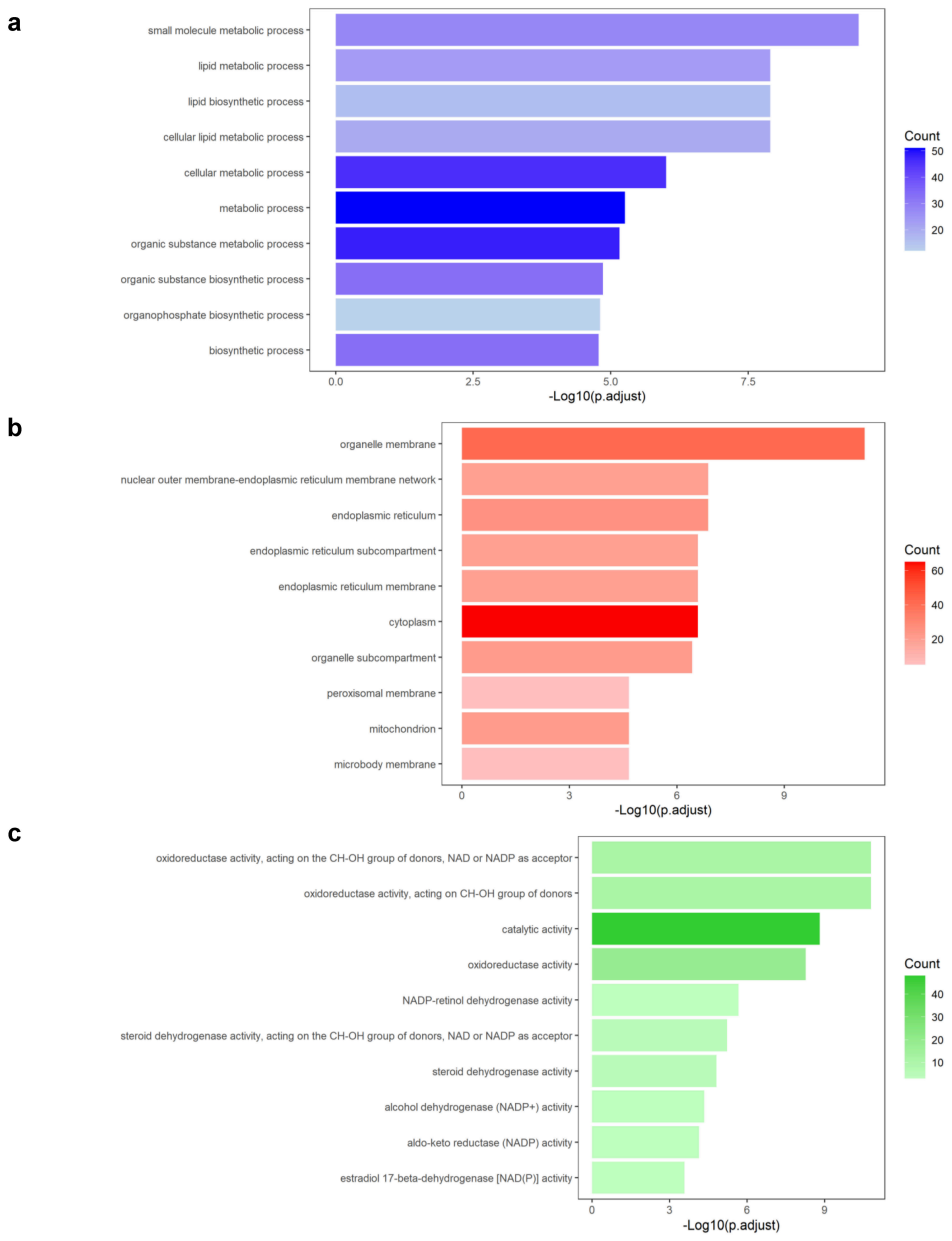
GO enrichment analysis of proteins downregulated in the TAC-treated group. Bar graphs show the 10 enriched GO terms most strongly associated with downregulated proteins in each GO category, based on adjusted *p*-values from enrichment analysis: (**a**) *Biological Process*, (**b**) *Cellular Component*, and (**c**) *Molecular Function*.

GO term enrichment analysis was performed to identify characteristic functional annotations associated with proteins that showed differential expression. Enrichment analyses were conducted for the three GO categories, *Biological Process*, *Cellular Component*, and *Molecular Function*. The 10 GO terms having the strongest associations with altered proteins, based on comparisons with database-registered GO terms, were visualized using bar charts (Figure 5). In addition, tree maps were generated to provide an overview of more granular GO terms (Figures S9–S11).

##### Biological Process

At the macroscopic level, *ribonucleoside bisphosphate metabolic process* (GO:0033875) was the top parent category. The 10 most enriched GO terms were predominantly composed of subcategories related to intermediate-level processes such as *lipid biosynthetic process* (GO:0008610), *monocarboxylic acid metabolic process* (GO:0032787), *organophosphate biosynthetic process* (GO:0090407), *cellular biosynthetic process* (GO:0044249), *cellular metabolic process* (GO:0044237), *metabolic process* (GO:0008152), and *organic substance biosynthetic process* (GO:1901576). In addition, *fatty acid elongation* (GO:0030497) was included among enriched categories, with Hsd17b12 and Tecr identified as the contributing proteins (Figure S9).

##### Cellular Component

The top 10 enriched GO terms were largely comprised of subcategories *endoplasmic reticulum membrane* (GO:0005789), *mitochondrial membrane* (GO:0031966), *peroxisomal membrane* (GO:0005778), *organelle membrane* (GO:0031090), and *cytoplasm* (GO:0005737). Enriched categories also included *lipid droplet* (GO:0005811), which reflected the presence of lipid-droplet–associated proteins such as Aifm2, Cideb, Dhrs3, Hsd17b11, Nsdhl, and Rdh10 (Figure S10).

##### Molecular Function

The top 10 enriched molecular functions mainly included subcategories *oxidoreductase activity, acting on the CH–OH group of donors, NAD or NADP as acceptor* (GO:0016616) and *catalytic activity* (GO:0003824). Additionally, glutathione transferase activity was represented among enriched terms, corresponding to proteins such as Gsta4 and Gstt2 (Figure S11).

#### 2.3.2. Pathway Enrichment Analysis

**Figure 6:**
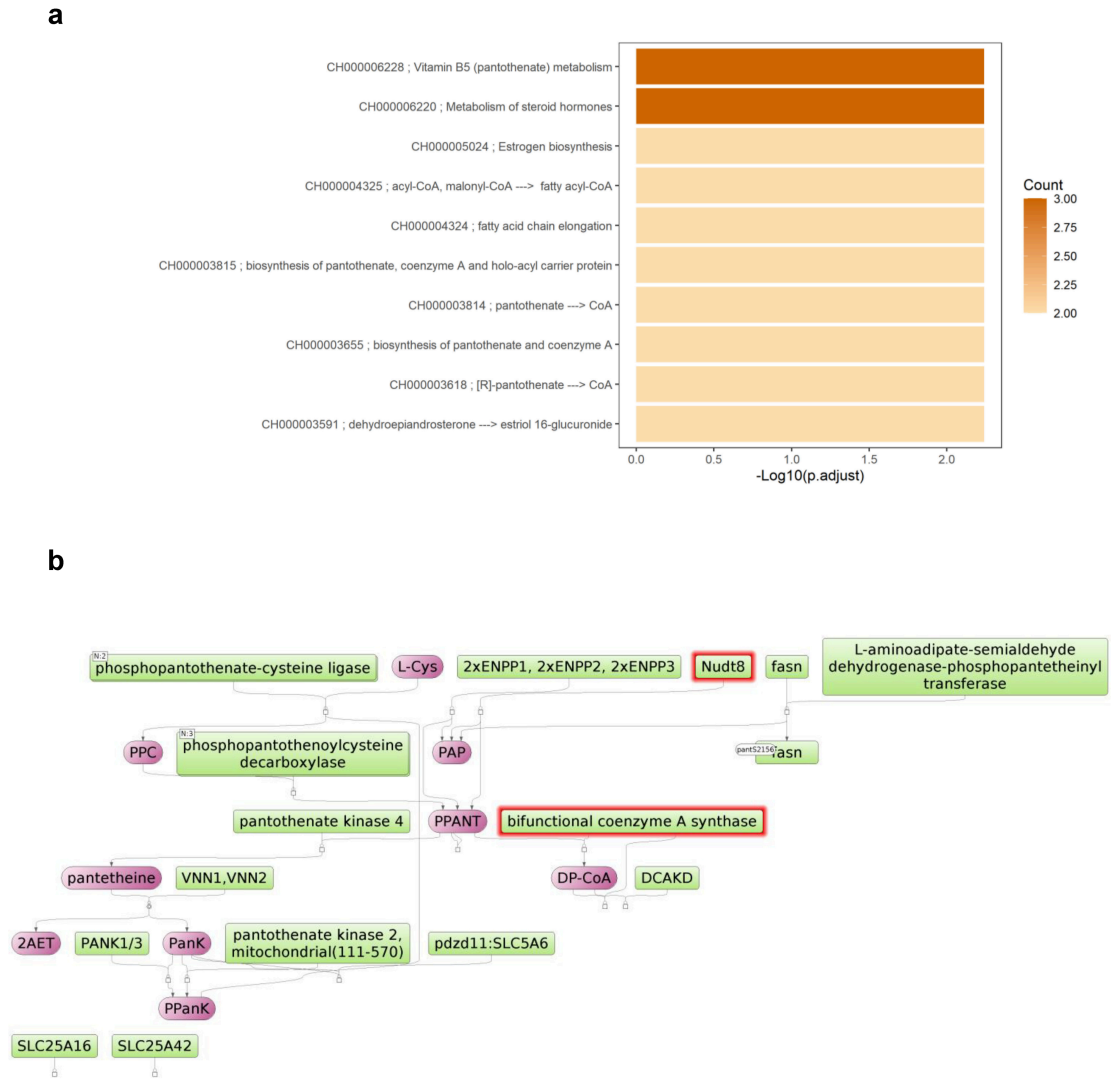
Pathway analysis of proteins downregulated in the TAC-treated group. (**a**) The 10 most enriched pathways identified by enrichment analysis are presented as a horizontal bar graph. Bars represent the enrichment score, and pathway names are displayed on the *y*-axis. (**b**) A representative pathway diagram illustrating key molecular interactions and components involved in TAC-responsive processes, with particular emphasis on *vitamin B5 (pantothenate) metabolism*, presented here as one of the representative downregulated pathways. In the diagram, green, rounded rectangles denote macromolecules, representing individual proteins, protein complexes, or related structures. Molecules outlined in red indicate those identified as significantly associated, based on the enrichment analysis. A capsule-shaped element positioned at the upper left of a green, rounded rectangle, labeled “pantS2156,” indicates phosphorylation at serine residue 2156 of pantothenate kinase. Pink capsule-shaped elements represent simple chemicals. Regarding edges, a black arrow indicates production, a double circle indicates a dissociation reaction, and a white-outlined square represents a reaction categorized as a process.

Pathway analysis was performed to characterize pathway-level alterations by mapping differentially expressed proteins onto curated pathways, including metabolic, signaling, and membrane transport pathways. Enrichment analysis was conducted using annotated pathway databases, and the top 10 enriched pathways were visualized using bar charts (Figure 6). In addition, the two most statistically significant pathways, *vitamin B5 (pantothenate) metabolism* and *estrogen biosynthesis*, ranked by ascending *p*-value, were illustrated separately to highlight their enrichment patterns (Figure 6 and Figure S12). Proteins contributing to these pathways included Nudt8 and bifunctional coenzyme A synthase for the vitamin B5 pathway, and estradiol 17β-dehydrogenase 2 (HSD17B2) and DHRS8 for the estrogen biosynthesis pathway.

#### 2.3.3. Disease Enrichment Analysis

Disease enrichment analysis of proteins downregulated by TAC treatment yielded several central nervous system (CNS)-related categories, including brain edema, stroke, and transverse myelitis (Figure S13). Appearance of these CNS-related disease terms should be interpreted as reflecting literature-based association patterns rather than activation of CNS-specific molecular programs in the kidney.

## 3. Discussion

These results provide clinically relevant insights into TAC-induced CKD. The principal finding of this study is that molecular abnormalities in TAC-treated kidneys can be clearly delineated along two major pathological axes: activation of the replication stress response and widespread suppression of lipid metabolism, mitochondrial function, and antioxidant defense. Although stringent detection (FDR < 0.01) identified only a limited number of upregulated and downregulated proteins, enrichment analyses incorporating proteins that met an FDR < 0.05 consistently highlighted these two molecular disease processes. Notably, the downregulated protein group contains molecules essential for renal homeostasis, including those involved in water and ion transport, epithelial barrier integrity, lipid and mitochondrial metabolism, and oxidative stress defense. These findings suggest that TAC treatment disrupts multiple aspects of the kidney’s fundamental stress-response network.

### 3.1. Identification of Differentially Expressed Proteins

A total of 7466 proteins were identified by LC-MS. After preprocessing in Perseus and applying conventional proteomic thresholds (*p* < 0.05 and a fold change ≥ 2 or ≤0.5), 27 proteins were upregulated, and 64 were downregulated, representing approximately 1.2% of the quantified proteome. In light of growing concerns regarding multiple testing in large-scale proteomics [12], permutation-based FDR correction was subsequently applied to obtain a more statistically robust set of differentially expressed proteins. Under a criterion of FDR < 0.05, 34 proteins were upregulated, and 90 were downregulated, whereas a stricter FDR < 0.01 threshold yielded a smaller subset of high-confidence alterations. These proteins represent the most consistent changes induced by TAC, whereas the broader FDR < 0.05–based dataset (Section 4.2 and Section 4.3) was used for pathway-level analyses.

Among these high-confidence proteins, several were particularly informative for understanding mechanisms of TAC-induced kidney injury. KAT6A, a lysine acetyltransferase that modifies histone lysine residues and contributes to chromatin organization and transcriptional regulation [13], was upregulated in TAC-treated kidneys. Histone acetylation is a key epigenetic mechanism that orchestrates transcriptional reprogramming during epithelial repair, and recent, single-nucleus, multi-omic studies have demonstrated that chromatin remodeling differentiates successful from failed repair during the transition from acute kidney injury (AKI)-to-CKD [14]. Thus, upregulation of KAT6A likely reflects an injury-induced epigenetic response and may also relate to its known functions in inflammatory and fibrotic gene regulation [15].

NCKAP1, a core subunit of the WAVE regulatory complex involved in actin cytoskeleton remodeling, was also upregulated. NCKAP1 is essential for immune cell migration and actin dynamics, and its deficiency results in impaired neutrophil and T-cell motility [16]. Upregulated NCKAP1 expression has also been implicated in fibrosis progression in renal ischemia–reperfusion injury models [17]. Therefore, its upregulation in TAC-treated kidneys likely reflects activation of cytoskeletal remodeling pathways triggered by tubular epithelial injury.

In contrast, several downregulated proteins provided insight into immunosuppressive mechanisms and mitochondrial dysfunction. IGHG2A, encoding the constant region of the IgG2a isotype, was downregulated following TAC administration. By inhibiting calcineurin–NFAT signaling, TAC suppresses IL-2 and IFN-γ production, thereby reducing IFN-γ–dependent class-switch recombination. Consistent with earlier reports showing that FK506 decreases circulating and glomerular IgG2a levels in NZB/W F1 mice [18], the downregulation observed here aligns with known immunosuppressive actions of TAC.

NDUFC2, an accessory subunit of mitochondrial complex I, was also downregulated. Reduced NDUFC2 expression impairs oxidative phosphorylation, diminishes ATP generation, and increases electron leakage, resulting in excessive reactive oxygen species (ROS) production [19,20]. NDUFC2 deficiency is a known cause of Leigh syndrome [21], and decreased NDUFC2 is linked to mitochondrial dysfunction, inflammation, and renal abnormalities in hypertensive rodent models and human genetic studies [22]. TAC-induced chronic nephrotoxicity shares these mechanistic features: decreased mitochondrial membrane potential, ATP depletion, and ROS overproduction [7]. Thus, NDUFC2 downregulation reinforces a shared pathogenic cascade: mitochondrial dysfunction → energy depletion → oxidative stress → cell death → inflammation/fibrosis, suggesting that strategies aimed at preserving NDUFC2-dependent mitochondrial function may mitigate TAC-induced renal injury.

Taken together, KAT6A (upregulated), NCKAP1 (upregulated), and NDUFC2 (downregulated) represent key molecular signatures that capture three major mechanistic axes of TAC-induced chronic nephrotoxicity: epigenetic remodeling, cytoskeletal regulation, and mitochondrial homeostasis. In kidney injury, KAT6A, a histone acetyltransferase, is central to epigenetic regulation, and abnormalities in histone modifications and epigenetic “memory” have been implicated in progression of renal disease. Potential efficacy of epigenetic drugs, including histone deacetylase inhibitors, has also been suggested in this context [23]. With KAT6A/B inhibitors, such as PF-07248144 [24], already advancing in clinical trials for cancer, therapeutic repurposing for kidney disease is theoretically feasible.

NCKAP1, which regulates actin cytoskeletal dynamics and contributes to cell migration and tissue remodeling, suppresses tumor progression in renal cancer via the PI3K/AKT/mTOR pathway, suggesting that cytoskeletal stabilization may also serve in renoprotection [25].

Conversely, downregulation of NDUFC2 reportedly impairs mitochondrial complex I function, leading to ATP depletion, increased ROS production, and heightened susceptibility to cellular stress. Although direct evidence linking NDUFC2 deficiency to cell death or fibrosis is limited, loss of another complex I subunit, NDUFS4, induces severe mitochondrial dysfunction that contributes to tissue injury and disease progression [26].

These three molecules, KAT6A, NCKAP1, and NDUFC2, may represent candidate therapeutic targets and biomarkers that warrant further investigation in TAC-induced kidney injury. A multilayered therapeutic strategy integrating epigenetic modulation, cytoskeletal stabilization, and mitochondrial protection may offer a novel avenue for treating kidney diseases.

### 3.2. Bioinformatic Analyses of Proteins Upregulated by TAC

#### 3.2.1. GO Enrichment Analysis

Proteins upregulated by TAC treatment exhibited strong enrichment in GO categories related to *DNA replication* and *DNA damage repair*. In particular, core components required for replication fork formation, including the MCM complex and the Cdc45–MCM–GINS (CMG) helicase complex, were significantly overrepresented [27,28]. Consistent with this, *Molecular Function* terms such as *helicase activity* and *origin DNA binding* ranked among the top annotations. These results suggest that TAC-induced cellular stress activates DNA damage response (DDR) and replication-stress pathways, leading to increased expression of proteins involved in cell-cycle progression.

In addition to DNA-related processes, the *Biological Process* term *copper ion detoxification* was prominently enriched, reflecting the presence of metallothioneins Mt1 and Mt2. This finding indicates activation of metal-ion–associated stress responses following TAC exposure [29].

Furthermore, enrichment of collagen metabolic processes in *Biological Process*, along with Cellular Component terms such as *collagen-containing extracellular matrix* and *external side of plasma membrane*, suggests alterations in extracellular matrix (ECM) composition and membrane-associated signaling. These features may represent early molecular events associated with tissue remodeling or fibrotic activation. *Molecular Function* terms included *integrin binding* and *lipid binding*, contributed by proteins such as Mfge8, Vcam1, Aldh1a2, Bspry, and Cadps2.

Among these, Mfge8 is a secreted glycoprotein that recognizes phosphatidylserine on apoptotic cells and promotes its clearance via binding to αVβ3/β5 integrins through its RGD motif [30]. Notably, αVβ3 and αVβ5 integrins are well known for their roles in angiogenesis [31], suggesting that Mfge8 may contribute not only to apoptotic-cell clearance, but also to tissue repair and remodeling. Based on our previous findings involving αVβ6 integrin [10], Mfge8 may further participate in TGF-β activation and ECM remodeling, functioning as an early molecular switch that initiates fibrotic programs.

#### 3.2.2. Pathway Enrichment Analysis

Pathway enrichment results provide important mechanistic insights into how TAC perturbs cellular homeostasis. Marked enrichment of pathways related to G1/S transition, S phase progression, G2/M checkpoints, and mitotic entry suggests that TAC induces substantial disturbances in cell-cycle regulation. Concurrent enrichment of DNA strand elongation, DNA synthesis, and pre-replicative complex assembly further indicates that TAC-exposed cells experience sustained pressure on DNA replication machinery, an effect commonly observed under replication-stress conditions.

A particularly notable finding was enrichment of the pathway *Activation of ATR in response to replication stress*. ATR is a central serine/threonine kinase in the canonical DDR that senses replication-fork stalling and accumulation of single-stranded DNA, subsequently phosphorylating CHK1 to initiate checkpoint activation and facilitate DNA repair. Engagement of this ATR-dependent surveillance pathway suggests that TAC-induced metabolic or oxidative alterations may overload the replication apparatus, triggering an adaptive DDR response.

Importantly, chronic replication stress drives cellular senescence [32], and the senescence-associated secretory phenotype creates a pro-fibrotic microenvironment through release of inflammatory cytokines, chemokines, and growth factors. This aligns with findings from ATR-deficient mouse models, in which multiple forms of renal injury, including cisplatin nephrotoxicity, ischemia–reperfusion injury, and obstructive nephropathy, result in exacerbated DNA damage, enhanced G_2_/M checkpoint arrest, accelerated fibrosis, and worsened renal dysfunction. These studies demonstrate that ATR-mediated DDR is essential for suppressing renal fibrogenesis [33].

Although enrichment of MCM-, CMG-, and ATR-related proteins is consistent with activation of replication-stress responses, alternative explanations should also be considered. Increased abundance of these proteins may reflect cell-cycle re-entry during tissue repair, changes in cellular composition, or proliferation of inflammatory and stromal cells in response to chronic injury. Therefore, while the present proteomic findings support activation of pathways associated with replication stress, additional studies will be required to determine the relative contribution of these biological processes in TAC-induced chronic nephrotoxicity.

In the context of TAC-induced chronic nephrotoxicity, activation of these pathways suggests that metabolism-driven replication stress may serve as an upstream trigger of fibrotic remodeling, acting in parallel with established mechanisms such as oxidative stress, mitochondrial dysfunction, and epithelial injury. These pathway signatures therefore broaden the mechanistic framework of TAC toxicity beyond calcineurin inhibition, highlighting replication stress and DDR dysregulation, as previously underappreciated contributors to renal injury and fibrosis.

#### 3.2.3. Upstream Enrichment Analysis

Upstream regulator analysis identified several transcriptional and signaling factors previously implicated in fibrotic progression. Among them, ARNTL (also known as BMAL1) emerged as a notable candidate. BMAL1 is a core component of the circadian clock that forms a heterodimer with CLOCK to regulate timing of cell-cycle progression and metabolic rhythms. Recent studies have demonstrated that BMAL1 contributes to DNA repair and replication-stress responses [34], and that its deficiency enhances TGF-β signaling and accelerates tissue fibrosis [35]. These findings suggest that circadian disruption may couple metabolic stress with chronic inflammation and fibrogenesis, positioning ARNTL/BMAL1 as a potential upstream driver of TAC-related injury.

Another regulator identified was Runx1, a transcription factor essential for hematopoietic lineage specification and fibroblast differentiation. Runx1 interacts with the TGF-β pathway to promote collagen synthesis and ECM accumulation. In this analysis, Runx1 was associated with pro-apoptotic factors such as Bim and Bax, histone acetylation mediated by MYST3, PKCγ-dependent signaling, and even circadian regulators such as CLOCK. Such a network implies that Runx1 may integrate replication-stress responses, programmed cell death, and circadian misalignment to drive fibrotic remodeling. Consistent with this interpretation, increased Runx1 expression has been reported in both cardiac and renal fibrosis models, and has gained attention as a potential anti-fibrotic therapeutic candidate [36].

Cdk5, traditionally recognized as a neuronal kinase, was also implicated as an upstream regulator. Although Cdk5 is generally activated by p35 and functions outside the canonical cell cycle [37], accumulating evidence indicates roles in DDR [38], cell migration, and inflammatory signaling [39]. Recent findings demonstrate that Cyclin G1 activates Cdk5 through a non-p35 pathway, promoting fibroblast activation and ECM production. Furthermore, pharmacologic inhibition of Cdk5 has shown anti-fibrotic efficacy in renal fibrosis models, indicating that the Cyclin G1–Cdk5 axis may represent a candidate therapeutic target in fibrotic diseases [40].

Collectively, these upstream factors, ARNTL, Runx1, and Cdk5, share mechanistic links through cell-cycle regulation, DDR signaling, and inflammatory pathways, suggesting that they may function as key nodes connecting TAC-induced replication stress with activation of fibrotic programs. Their involvement provides mechanistic insight into how metabolic and replication-related perturbations induced by TAC can converge to promote renal fibrosis.

#### 3.2.4. Disease Enrichment Analysis

Although several tumor-related disease terms emerged, these reflect universal proliferation and replication-stress signatures rather than tumor-type specificity. Associated genes, such as MCM2–7, AKT3, NES, and VCAM1, are core regulators of cell-cycle progression and replication-stress responses. This pattern aligns with pathway-level enrichment for G1/S transition, DNA strand elongation, and ATR-mediated replication-stress activation.

#### 3.2.5. Brief Summary of Upregulated-Protein Analyses

Taken together, results of enrichment analyses indicate that TAC exposure activates a broad spectrum of DNA replication–related pathways and triggers an ATR-dependent replication-stress response. While replication stress is a recognized feature of CKD pathology [33], our findings suggest that in TAC-induced chronic nephrotoxicity, drug-specific metabolic disturbances and oxidative stress may further exacerbate this response. Consistent with this interpretation, our metabolomic analysis previously demonstrated enhanced cytosine catabolism, a signature of increased nucleic-acid degradation, which is mechanistically congruent with elevated replication stress and impaired genome maintenance under TAC exposure [8]. Harmonizing with this view, TAC reportedly impairs antioxidant defense systems, including glutathione and superoxide dismutase, while promoting generation of ROS [41]. Moreover, our renal metabolomic analysis demonstrated that N-acetylcysteine (NAC) and other antioxidant metabolites were decreased in TAC-treated kidneys, indicating reduced endogenous antioxidant capacity. Although NAC itself was not tested as a rescue treatment, its depletion supports the interpretation that TAC-induced metabolic alterations diminish key redox-regulating metabolites, reinforcing oxidative stress as a central driver of TAC-induced chronic nephrotoxicity [7]. In addition, models of chronic TAC-induced kidney injury have shown NAD^+^ depletion and impairment of the salvage pathway, suggesting that metabolic dysfunction and oxidative stress synergistically amplify replication stress [8]. Together, these findings support a mechanistic model in which oxidative stress serves as a primary upstream driver, intensifying ATR activation and replication-stress pathways, which are also characteristic of CKD, but become more pronounced under TAC exposure due to overlapping redox and metabolic disturbances. This integrated view provides a coherent framework for understanding how TAC-induced oxidative injury converges with replication-stress signaling to promote DNA damage, maladaptive repair, and progression toward renal fibrosis.

### 3.3. Bioinformatic-Based Enrichment Analysis of TAC-Downregulated Proteins

#### 3.3.1. GO Enrichment Analysis

GO enrichment analysis of proteins downregulated by TAC revealed a prominent suppression of lipid-related *Biological processes*, particularly lipid biosynthesis and fatty-acid elongation. Downregulation of Hsd17b12 and Tecr, both of which are essential enzymes for elongation of long-chain and unsaturated fatty acids, suggests impaired membrane-lipid remodeling after TAC exposure.

In *Cellular Component* categories, proteins localized to the endoplasmic reticulum membrane, mitochondrial membrane, peroxisomal membrane, and lipid droplets were notably reduced, including Aifm2, Cideb, Dhrs3, Dhrs4, Hsd17b11, Hsd17b12, Nsdhl, and Rdh10. These proteins are significant in fatty-acid elongation, sterol and retinoid metabolism, neutral-lipid storage, and oxidative-stress responses, indicating a decline in both membrane-lipid remodeling capacity and lipid peroxidation defense.

*Molecular Function* terms were characterized by a marked reduction in NAD(P)^+^-dependent oxidoreductase activity, reflecting suppression of redox reactions required for lipid, steroid, and retinoid metabolism. Such decreases may reduce the cellular supply of long-chain and unsaturated fatty acids, with potential consequences for membrane fluidity and lipid-dependent signaling pathways. In addition, downregulation of Gsta4 and Gstt2, both involved in glutathione-dependent detoxification of lipid peroxides, suggests weakening of the glutathione-based lipid-peroxidation defense system. Concomitant suppression of both NAD(P)^+^-dependent and glutathione-dependent pathways raises the possibility that disruption of redox balance increases susceptibility to ferroptosis.

Overall, downregulation of lipid-biosynthetic enzymes and oxidoreductases involved in redox regulation is consistent with TAC-induced alterations in triglyceride composition revealed by our lipidomic analysis, specifically, the decrease in triglycerides with shorter carbon chains and greater saturation. These proteomic alterations align with our previous findings of elevated lactate in renal metabolomics [7], reduced NAD^+^ levels in plasma metabolomics [8], and TG compositional remodeling [9], indicating that proteome-level shifts observed here provide molecular support for TAC-induced metabolic dysfunction.

Among downregulated proteins, downregulation of Aifm2 (also known as FSP1; ferroptosis suppressor protein 1) is particularly noteworthy. FSP1 functions as an auxiliary antioxidant system that suppresses lipid-radical propagation by reducing CoQ_10_, thereby elevating the cellular threshold for iron-dependent lipid peroxidation [42]. This pathway operates independently of GPX4, which detoxifies lipid hydroperoxides using glutathione (GSH), and together they form a parallel, multilayered, antioxidant defense system. Thus, reduction in FSP1 implies weakening of a defense layer that acts independently of the GPX4–GSH axis.

Moreover, concurrent reduction in Gsta4 and Gstt2 further indicates attenuation of glutathione-mediated lipid-peroxide detoxification. Overlap of these changes suggests that multiple lipid-peroxidation defense systems, CoQ_10_–FSP1, GPX4–GSH, and GST-mediated detoxification, may be simultaneously compromised following TAC treatment.

In summary, these findings suggest that TAC shifts renal cells toward a state in which both the CoQ_10_–FSP1 axis and the GPX4–GSH axis are weakened, creating a cellular environment increasingly prone to lipid peroxidation. While these alterations support a heightened susceptibility to ferroptosis, they do not, by themselves, confirm that ferroptosis is occurring. Rather, they provide a molecular backdrop that could predispose TAC-treated cells to ferroptotic death under additional stress conditions.

#### 3.3.2. Pathway Enrichment Analysis

The present pathway analysis of TAC-downregulated proteins highlighted a prominent reduction in the vitamin B5 (pantothenate)–coenzyme A (CoA) biosynthetic pathway, suggesting a central metabolic bottleneck in renal tubular cells. CoA is an indispensable metabolic hub that links fatty-acid β-oxidation, the tricarboxylic acid (TCA) cycle, ketone-body and amino-acid oxidation, lipid synthesis, and even histone acetylation. Therefore, downregulation of CoA-synthesizing enzymes, most notably bifunctional coenzyme A synthase (COASY), is likely to restrict the intracellular CoA pool, thereby limiting formation of acyl-CoA and acetyl-CoA. Such alterations would collectively impair mitochondrial oxidative metabolism, resulting in slowed β-oxidation, reduced availability of TCA-cycle substrates, diminished ATP production, and increased strain on the electron transport chain, ultimately culminating in excessive ROS generation. Renal proximal tubules depend heavily on fatty-acid oxidation (FAO) as their primary energy source. Thus, impairment of mitochondrial oxidative metabolism in this segment readily precipitates early oxidative stress and mitochondrial dysfunction. This interpretation is consistent with the TAC-induced chronic nephrotoxicity Adverse Outcome Pathway described in the NDT 2025 supplement [43], in which disruption of metabolic homeostasis may accelerate ROS-driven mitochondrial injury, energy depletion, and lipotoxicity, thereby destabilizing the tubulointerstitial microenvironment.

Synergistic deterioration involving the carnitine axis is also mechanistically plausible. Our previous metabolomic analysis demonstrated marked carnitine depletion in TAC-treated mouse kidneys [7]. When combined with the CoA deficiency indicated in the present proteomic dataset, these findings imply dual bottlenecks: (1) impaired fatty-acid entry into mitochondria via the CPT1/2 system and (2) reduced acyl-CoA formation, both of which would hinder β-oxidation at multiple steps. The resulting cascade, lipid accumulation, increased ROS formation, and diminished ATP production further weaken the tubule–interstitium unit. This aligns with previous reports showing that L-carnitine supplementation alleviates oxidative stress, mitochondrial injury, and tubular cell death in renal injury models [44]. These data collectively support a therapeutic rationale for interventions that enhance fatty-acid oxidation, e.g., carnitine repletion, alongside strategies that restore CoA pools, e.g., pantothenate supplementation.

Among steroid-related pathways, downregulation of HSD17B2 is notable. HSD17B2 is a member of the short-chain dehydrogenase/reductase (SDR) family, and like its paralog, HSD11B2, catalyzes NAD^+^-dependent steroid inactivation. Reduced HSD17B2 expression may weaken steroid-inactivation capacity in the distal nephron and collecting duct, thereby contributing to diminished mineralocorticoid receptor (MR) protection. This reduction, together with DHRS8 downregulation, indicates broader suppression of NAD^+^-dependent steroid-detoxification pathways. Under these conditions, cortisol, which is normally excluded from MR activation through HSD11B2-mediated oxidation, may increasingly occupy and activate MR, a phenomenon well described in epithelial physiology [45].

Such inappropriate MR activation promotes sodium retention, disrupts ionic homeostasis, induces pro-inflammatory signaling, and amplifies TGF-β–driven fibrogenesis. These mechanisms mirror observations in kidney-specific Hsd11b2 knockout models, in which overactivation of epithelial sodium channels (ENaC) and Na-Cl cotransporters (NCC) leads to sodium retention, hypokalemia, salt-dependent hypertension, and renal fibrosis [46]. Importantly, TAC-treated kidneys already exhibit metabolic stress characterized by CoA depletion, mitochondrial dysfunction, and increased ROS. These metabolic derangements shift the intracellular redox environment toward a higher NADH/NAD^+^ ratio, creating a reductive state that we define as “NADH/NAD^+^ reductive stress.” Although reductive NADH/NAD^+^ shifts have been described in other pathological contexts, our prior work in fibroblasts from patients with Leigh syndrome and in complex I–deficient mouse models similarly demonstrated an elevated NADH/NAD^+^ ratio accompanied by mitochondrial injury, consistent with this framework [47]. This reductive imbalance diminishes the activity of NAD^+^-dependent enzymes such as HSD17B2 and DHRS8, thereby linking metabolic dysfunction to impaired steroid metabolism in TAC-induced nephropathy.

Collectively, these findings support a potential metabolism–endocrine amplification loop, in which TAC-induced metabolic stress disrupts mitochondrial oxidative metabolism and redox homeostasis, resulting in decreased steroid-inactivation capacity, inappropriate MR activation, and further mitochondrial injury. This interconnected cascade, metabolic stress → impaired steroid metabolism → MR hyperactivation → worsening oxidative and mitochondrial dysfunction, may sensitize kidneys to TAC-induced fibrotic remodeling and may be exacerbated under conditions such as low-sodium intake, which augments the renin–angiotensin–aldosterone system (RAAS) and MR signaling.

#### 3.3.3. Disease Enrichment Analysis

CNS-related disease terms identified for downregulated proteins do not indicate activation of CNS-specific programs. Instead, they reflect suppression of fundamental homeostatic pathways shared among organs, including solute and water transport, epithelial-barrier integrity, mitochondrial metabolism, and antioxidant defense. Such cross-organ stress signatures resemble those observed in tissues exposed to ischemia and oxidative injury, explaining the appearance of CNS-related categories.

#### 3.3.4. Brief Summary of Downregulated-Protein Analyses

TAC-downregulated proteins were predominantly linked to essential metabolic and homeostatic processes, including lipid metabolism, redox regulation, membrane-associated organellar function, CoA-dependent pathways, and hormone metabolism. Notably, multiple layers of lipid peroxidation defense, such as the fatty-acid elongation system, NAD(P)^+^-dependent redox enzymes, the GSH/GST system, and the FSP1/CoQ_10_ axis, were simultaneously reduced, suggesting a multilayered impairment of oxidative-stress control. These alterations align with the molecular framework of ferroptosis, a process increasingly recognized in kidney diseases, characterized by enhanced lipid peroxidation and vulnerabilities of the GPX4 and CoQ_10_ systems; thus, TAC-induced metabolic stress may sensitize tubular cells to ferroptosis [48].

Furthermore, suppression of the vitamin B5/CoA biosynthetic pathway restricts multiple metabolic nodes, including acyl-CoA formation, β-oxidation, and the TCA cycle, leading to mitochondrial dysfunction and increased ROS production. Such metabolic limitations profoundly impair fatty-acid oxidation–dependent energy metabolism of proximal tubules and are consistent with ischemia-like metabolic compromise and exacerbated oxidative stress, both of which represent central pathological features of TAC-induced chronic nephrotoxicity.

Our analysis revealed downregulation of multiple lipid-metabolizing enzymes, consistent with our previous observation of reduced free carnitine and acylcarnitines in TAC-treated kidneys [7]. This pattern also agrees with earlier reports showing TAC-induced alterations in triglyceride composition, shorter chain length and lower unsaturation, and elevated lipid oxidative stress [9]. Disruption of the lipid metabolic network likely contributes to a sequential cascade: carnitine depletion → impaired β-oxidation → increased lipid peroxidation.

Additionally, reduced levels of steroid-inactivating enzymes (HSD17B2, DHRS8, etc.) may potentiate inflammation and fibrosis via aberrant MR activation. Metabolic stress in TAC-treated kidneys can destabilize NADH/NAD^+^ reductive stress, thereby further diminishing activity of NAD^+^-dependent enzymes.

Taken together, TAC-downregulated proteins reflect a molecular landscape in which multiple pathological drivers, ischemia-like metabolic failure, lipid peroxidation, compromised antioxidant defense, membrane dysfunction, and MR overactivation, progress concurrently. These features exhibit strong concordance with established mechanisms of TAC-induced chronic nephrotoxicity, including ischemia, metabolic disruption, oxidative stress, epithelial injury, and fibrosis, and collectively represent a core molecular signature of TAC-related kidney injury.

### 3.4. Overall Integrated Summary and Interpretation

In this study, we conducted a dedicated protein-centered investigation using a previously generated proteomic dataset from a TAC nephrotoxicity model to complement our previous metabolomic, lipidomic, and trans-omic investigations. Unlike our previous metabolomic and lipidomic studies, which primarily identified alterations in metabolites and lipid species, and unlike our previous trans-omic study, which focused on transcriptome–proteome integration and upstream regulator prediction, the present study was specifically designed to characterize biologically meaningful protein-level alterations through a dedicated protein-centered analytical strategy. This approach enabled identification of protein signatures associated with the FSP1/CoQ_10_ antioxidant system, GST-related redox regulation, CoA metabolism, mitochondrial function, and replication-stress pathways that were not comprehensively evaluated in our previous studies. These findings extend our previous metabolomic and lipidomic observations by providing a protein-level framework linking altered lipid metabolism, redox regulation, mitochondrial function, and replication-stress-related pathways. As a result, we successfully identified protein-level alterations that were not evident at the mRNA level, including upregulation of KAT6A and NCKAP1 and downregulation of NDUFC2 and several fatty-acid-elongating enzymes. Bioinformatic analyses based on these differentially expressed proteins revealed two major pathological axes characteristic of TAC-induced nephropathy: enhanced DNA-replication stress responses in upregulated proteins, and impaired lipid/energy metabolism together with weakened antioxidant defenses in downregulated proteins. These findings provide complementary biological insights beyond those obtained from our previous trans-omic study by systematically characterizing protein-level molecular alterations. Notably, this study provides proteome-level evidence of substantive dysfunction in membrane lipid remodeling, CoA-dependent pathways, and the ferroptosis suppressor protein 1 (FSP1)/CoQ_10_ system, alterations difficult to detect using mRNA-centered approaches alone.

Disease enrichment analysis further demonstrated clear distinctions between the two protein groups. The upregulated set was characterized by tumor- and proliferation-related terms, most prominently medulloblastoma and mesothelioma, which reflect universal proliferation and replication-stress signatures associated with factors such as MCM2–7, AKT3, and NES rather than true oncogenic specificity.

In contrast, downregulated proteins showed enrichment for CNS-associated disease terms, including brain edema, stroke, optic neuritis, and transverse myelitis. These terms do not imply that the kidney engages CNS-specific molecular programs. Instead, they represent cross-disease signatures arising from concurrent declines in core homeostatic pathways shared across multiple organs, such as water and ion transport, epithelial-barrier maintenance, mitochondrial metabolism, and antioxidant defense. The appearance of CNS-related terms therefore reflects universal stress-response patterns, ischemia, oxidative stress, inflammatory injury, and barrier dysfunction, rather than organ-specific pathology.

Together, these findings suggest that TAC nephropathy shares molecular features with biological programs described in various extra-renal diseases. These disease-enrichment findings should be regarded as exploratory observations that may provide insight into molecular pathways shared among different diseases and organ systems. Such observations may help generate hypotheses regarding whether mechanisms identified in fields such as oncology and neurology can offer insights into TAC-induced chronic nephrotoxicity. In particular, therapeutic approaches developed for disorders involving mitochondrial dysfunction or lipid peroxidation may be conceptually relevant to TAC nephropathy, which exhibits impairment of the NDUFC2–CoQ–FSP1 axis.

Multiple pathogenic mechanisms underlying TAC nephropathy, such as vasoconstriction-driven ischemia due to reduced NO and elevated endothelin, lipid metabolic impairment, ferroptosis triggered by diminished antioxidant capacity, and TGF-β–dependent fibrosis, have previously been proposed and were recently summarized in an NDT 2025 Supplement [43]. Proteomic alterations identified here show strong concordance with these classical mechanisms and reinforce established pathogenic frameworks at the protein-phenotype level.

Furthermore, the observed reduction in NDUFC2 suggests impaired electron transfer at the ubiquinone-binding site of mitochondrial respiratory complex I, thereby lowering the reduction capacity of the CoQ pool and functionally linking it to weakened lipid-peroxidation suppression via the FSP1/CoQ_10_ axis, as supported by previous reports [49]. Specifically, the sequence NDUFC2 reduction → decreased complex I activity → NADH/NAD^+^ imbalance → restricted CoQH_2_ generation → enhanced lipid peroxidation provides an integrated redox cascade that may exacerbate antioxidant failure in TAC nephropathy.

Importantly, this concept aligns with our previous findings in fibroblasts from patients with Leigh syndrome and in complex I-deficient mouse models [47], wherein complex I impairment shifts the intracellular redox environment toward a more reduced state, with augmented mitochondrial damage and lipid peroxidation, all strongly correlated with disease severity. These observations support a conserved pathogenic chain, “complex I failure → reductive NADH/NAD^+^ shift → lipid peroxidation”, that spans tissues and disease contexts.

Collectively, our findings demonstrate that TAC nephropathy is characterized by concurrent activation of replication-stress pathways and suppression of lipid/energy metabolism and multilayered antioxidant systems, thereby establishing a molecular foundation that exacerbates oxidative stress, inflammation, and fibrosis. This study substantially advances the molecular understanding of TAC-induced chronic nephrotoxicity by providing protein-level phenotypes not captured by mRNA-centered analyses. Many molecular features discovered here diverge from mRNA expression profiles and are visualized only through proteomic approaches, underscoring the importance of protein-level analyses in identifying molecular pathways potentially involved in disease pathogenesis and candidate therapeutic targets.

### 3.5. Limitations

This study has several limitations. First, it was specifically designed as a protein-centered investigation and did not perform additional multi-omic integration. Although transcriptomic, metabolomic, lipidomic, and trans-omic datasets have previously been generated using this model of TAC-induced chronic nephrotoxicity, the objective of the present study was to characterize biologically meaningful protein-level alterations independently. Therefore, potential discrepancies between mRNA and protein levels, as well as direct causal links with metabolites, remain unresolved. Second, our analysis was limited to the renal cortex, and spatial variations in the medulla or among tubular segments were not assessed. However, because renal fibrosis predominantly occurs in the cortex, this choice is considered appropriate for investigating these pathological processes. Temporal changes from the acute to chronic phases also remain to be clarified. Third, the pathogenic relevance of identified proteins requires further functional validation through genetic or pharmacological approaches. Moreover, as this study relied on an animal model, caution is warranted when extrapolating these findings to human nephropathy.

Finally, disease terms identified through disease enrichment represent statistical associations based on similarities in expression patterns. They do not directly imply diagnostic utility or therapeutic applicability for specific diseases. Therapeutic repurposing strategies discussed here should therefore be regarded as conceptual possibilities derived from shared molecular network features.

### 3.6. Future Perspectives

Although transcriptomic, metabolomic, lipidomic, and trans-omic analyses have previously been performed in this TAC nephropathy model, further integrative analyses incorporating replication-stress-associated proteomic signatures identified in the present study with these existing datasets may provide a more comprehensive understanding of the crosstalk between metabolic dysfunction and replication stress. Such analyses would complement the present protein-centered investigation by integrating proteome-specific biological alterations identified here with broader molecular networks characterized in our previous trans-omic study. In addition to characteristic proteins identified in this study, KAT6A, NDUFC2, Hsd17b12, and Tecr, key molecules representing the two major pathological axes (replication-stress and metabolic-impairment pathways), such as Aifm2 (FSP1) and Gsta4/Gstt2 (GST family), will require functional validation using genetically modified models or pharmacological inhibitors.

Furthermore, to enhance spatial resolution in the kidney, future research should combine medulla-focused and tubule segment–specific proteomics with imaging mass spectrometry to clarify spatial and temporal dynamics of disease progression. In addition, development of therapeutic strategies targeting these molecules and related metabolic pathways, such as epigenetic modulators, mitochondrial-protective agents, and lipid-metabolism–modifying drugs, as well as evaluation of their potential as biomarkers, will represent important challenges moving forward.

## 4. Materials and Methods

### 4.1. Proteomic Data and Ethical Statement

Proteomic data analyzed in this study were obtained from our previously established TAC-induced chronic nephrotoxicity mouse model [10]. Briefly, male C57BL/6J mice (8 weeks old) were assigned to control and TAC-treated groups (*n* = 5 per group). Mice were fed a 0.01% sodium diet from 7 weeks of age, and TAC administration (1 mg/kg/day) was initiated at 8 weeks of age. Renal cortex samples were collected and subjected to LC–MS/MS-based proteomic analysis.

Proteomic analysis was performed using an UltiMate 3000 RSLCnano LC system (Thermo Fisher Scientific, Bremen, Germany) coupled to a Q Exactive HF-X Hybrid Quadrupole-Orbitrap mass spectrometer (Thermo Fisher Scientific, Bremen, Germany) operated in data-independent acquisition (DIA) mode.

Quality control (QC) was performed using trypsin-digested HEK293 cell extracts measured at regular intervals (approximately one to two times per week). Instrument performance was monitored using the number of precursor ions and identified proteins. If QC criteria were not met, instrument maintenance and cleaning were performed before continuing sample analysis. Sample measurements were not randomized. The original study included animal experiments, proteomic measurements, and trans-omic analyses, whereas the objective of the present study was to perform a protein-centered investigation using the same proteomic dataset to characterize biologically meaningful protein-level alterations. No new animal experiments were performed in the present study. Detailed experimental procedures are described in our previous publication [10]. Raw LC–MS/MS data and processed protein matrices are available from the corresponding author upon request.

### 4.2. Data Processing

Raw data were processed using DIA-NN (version 1.9.1, https://github.com/vdemichev/DiaNN, accessed on 18 November 2024). An in silico–predicted spectral library was generated with DIA-NN based on the mouse protein sequence database (UniProt ID: UP000000589; reviewed, canonical; 21,709 entries; downloaded on 1 April 2024). The following search parameters were applied: trypsin as the protease, one missed cleavage, peptide length of 7–45 amino acids, precursor charge range of 2–4, precursor mass range of 500–740, fragment ion m/z range of 200–1800, and mass accuracy of 10 ppm. Carbamidomethylation of cysteine was set as a static modification.

Enabled options included “Heuristic protein interferences,” “Use isotopologues,” “MBR,” and “No shared spectra.” Additional commands used were “mass acc cal 10,” “peak translation,” and “matrix spec q.”

Protein identification thresholds were set at an FDR of <1% at both the peptide and protein levels.

### 4.3. Data Analysis Using Perseus

Data analysis was performed using Perseus v1.6.15.0 (https://maxquant.org/perseus/, accessed on 18 November 2024) [12].

Data were processed as follows:

(1) Protein intensity values were log_2_-transformed.
(2) Proteins quantified in at least 70% of samples in at least one comparison group were retained (proteins with excessive missing values across all groups were removed).
(3) Missing values, when present, were processed using the standard Perseus imputation workflow implemented in Perseus (width 0.3, downshift 1.8). Importantly, none of the proteins included in the final significant protein dataset required imputation, and all downstream biological analyses were performed using proteins with complete quantitative data across all samples.
(4) Fold changes, *p*-values (Student’s *t*-test), and Z-scores were calculated for each comparison using the imputed dataset.
(5) Differentially expressed proteins were selected using commonly applied proteomic thresholds (*p* < 0.05 and fold change ≥ 2), and volcano plots and heatmaps were generated.

Because quantitative proteomic analyses involve simultaneous statistical testing of a large number of proteins, false-positive findings arising from multiple hypothesis testing were controlled using the permutation-based FDR procedure implemented in Perseus. Proteins with FDR < 0.05 or FDR < 0.01 were considered statistically significant and constituted the primary dataset for downstream analyses.

### 4.4. Data Analysis Using the geneXplain Platform

Functional analysis of differentially expressed proteins was performed using the geneXplain platform (geneXplain GmbH, Wolfenbüttel, Germany; https://genexplain.com/, accessed on 16 October 2025). GO analysis, pathway analysis, upstream analysis, and disease enrichment analysis were conducted to identify enriched biological processes, signaling pathways, regulatory factors, and disease associations.

The geneXplain platform was selected because it allows integrated analysis of GO terms, signaling pathways, upstream regulators, and disease associations within a unified analytical framework. In addition, geneXplain provides access to multiple curated databases, including TRANSPATH, TRANSFAC, and HumanPSD, enabling comprehensive interpretation of proteomic alterations beyond conventional functional enrichment analysis.

For downstream bioinformatic analyses, identified mouse proteins were mapped to their corresponding gene symbols, and where required by the annotation databases, were mapped using geneXplain to the corresponding human orthologs.

#### 4.4.1. GO Analysis

GO analysis was performed to identify characteristic GO terms associated with differentially expressed proteins. In this analysis, the TRANSPATH database (geneXplain GmbH; accessed on 21 February 2025) was used to conduct enrichment analysis for three GO categories: *Biological Process*, *Cellular Component*, and *Molecular Function*. Enriched GO terms were listed, and bar charts and tree maps were generated for visualization.

#### 4.4.2. Pathway Enrichment Analysis

Pathway analysis was performed to identify characteristic metabolic, signaling, membrane transport, and other biological pathways associated with differentially expressed proteins. The TRANSPATH database (geneXplain GmbH; accessed on 21 February 2025) was used to extract significantly enriched pathways. Enriched pathways were listed, and bar charts were generated. In addition, the top two or three statistically most characteristic pathways were visualized.

#### 4.4.3. Upstream Analysis

Upstream analysis was performed using significantly altered proteins identified in the differential abundance analysis as input. The analysis was conducted using the TRANSFAC 2.0 database (geneXplain GmbH; accessed on 14 February 2025) and the TRANSPATH database (geneXplain GmbH; accessed on 21 February 2025) to identify potential upstream regulatory factors associated with the observed protein-expression changes.

For proteins that were upregulated in the TAC group, enriched upstream factors were listed, and bar charts were generated for visualization. In addition, upstream networks for the top three candidate factors were illustrated. Because no upstream factors showed statistically significant enrichment for proteins downregulated in the TAC group, upstream analysis was not performed for these proteins.

#### 4.4.4. Disease Enrichment Analysis

Disease enrichment analysis was conducted using the HumanPSD database (geneXplain GmbH; accessed on 20 February 2025), which catalogs proteins associated with various diseases. Differentially expressed proteins were analyzed to identify diseases with high relevance to these proteins. Enriched disease terms were listed, and bar charts were generated for visualization.

## 5. Conclusions

In conclusion, our comprehensive proteomic and bioinformatic analyses delineated the molecular landscape altered by TAC exposure and systematically identified distinct protein expression profiles associated with injury and metabolic dysfunction. In particular, this study revealed multilayered alterations in antioxidant defense systems, including the FSP1-mediated CoQ_10_ regeneration pathway and the GST–associated antioxidant system, suggesting that impairment of antioxidant defense contributes to the pathophysiology of TAC-induced chronic nephrotoxicity. Upregulated proteins highlighted activation of replication-stress and inflammation-related signaling, whereas downregulated proteins implicated impaired lipid metabolism, CoA biosynthesis, mitochondrial function, and redox regulation. Together, these findings suggest that TAC exposure is associated with activation of cellular stress responses accompanied by disruption of metabolic and antioxidant homeostasis. Protein networks identified here offer promising candidate therapeutic targets and biomarkers for future investigation in drug-induced kidney injury. Future mechanistic and in vivo studies will help to clarify the contribution of each pathway to disease progression. Furthermore, dysregulation of lipid metabolic pathways, mitochondrial function, and antioxidant systems may provide opportunities for development of protective strategies and precision diagnostics to enable safe clinical use of TAC. The proposed mechanisms require further experimental validation and should be regarded as hypotheses generated from proteomic analyses.

## Data Availability

The dataset is available from the corresponding author upon request.

## Supplementary Materials

The following supporting information can be downloaded at: https://www.mdpi.com/article/10.3390/ijms27157030/s1.
