# Supplementary material for: Proteomic Characterization of Replication Stress and Impaired Antioxidant Defense in Tacrolimus-Induced Chronic Nephrotoxicity
Source: Int J Mol Sci. 2026 Aug 5;27(15):7030. doi: 10.3390/ijms27157030 (PMC13466515; doi:10.3390/ijms27157030)
Supplement: Supplementary file 1 [file ijms-27-07030-s001.zip › ijms-4399670-supplementary.pdf]

Supplementary Figures

Figure S1

a

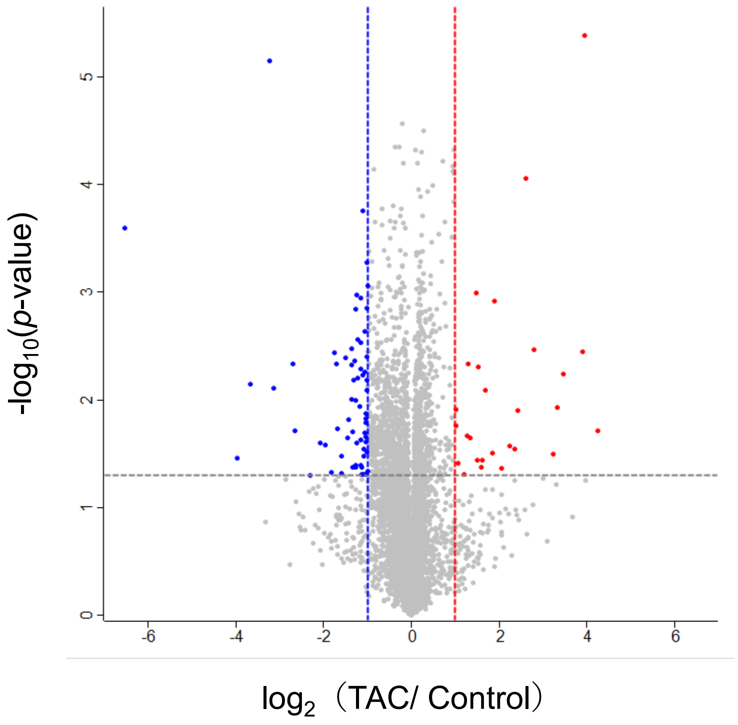

-log10(p-value)
log2 (TAC/ Control)

b

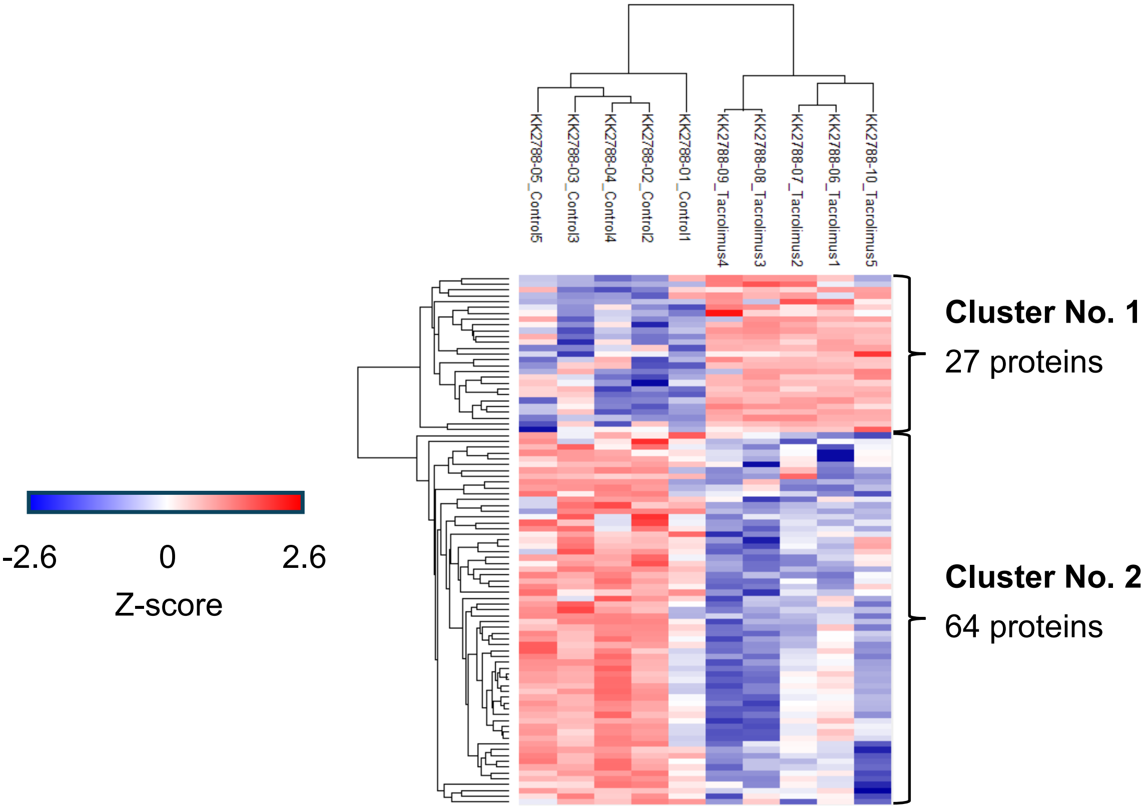

KK2788-05_Control5
KK2788-03_Control3
KK2788-04_Control4
KK2788-02_Control2
KK2788-01_Control1
KK2788-09_Tacrolimus4
KK2788-08_Tacrolimus3
KK2788-07_Tacrolimus2
KK2788-06_Tacrolimus1
KK2788-10_Tacrolimus5
-2.6
0
2.6
Z-score
Cluster No. 1
27 proteins
Cluster No. 2
64 proteins

Figure S2

a

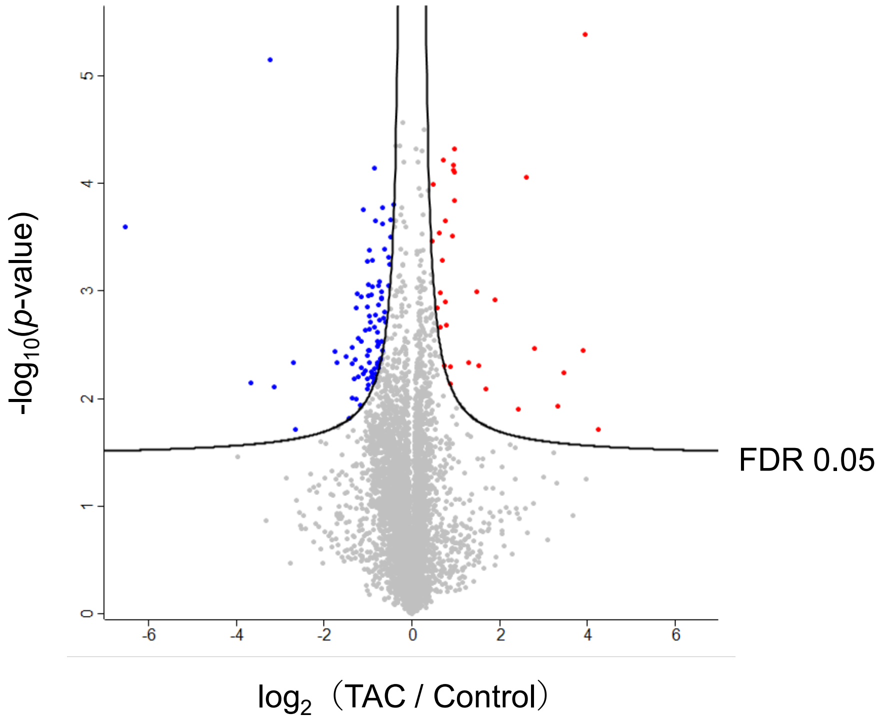

-log10(p-value)
FDR 0.05
log2（TAC / Control）

b

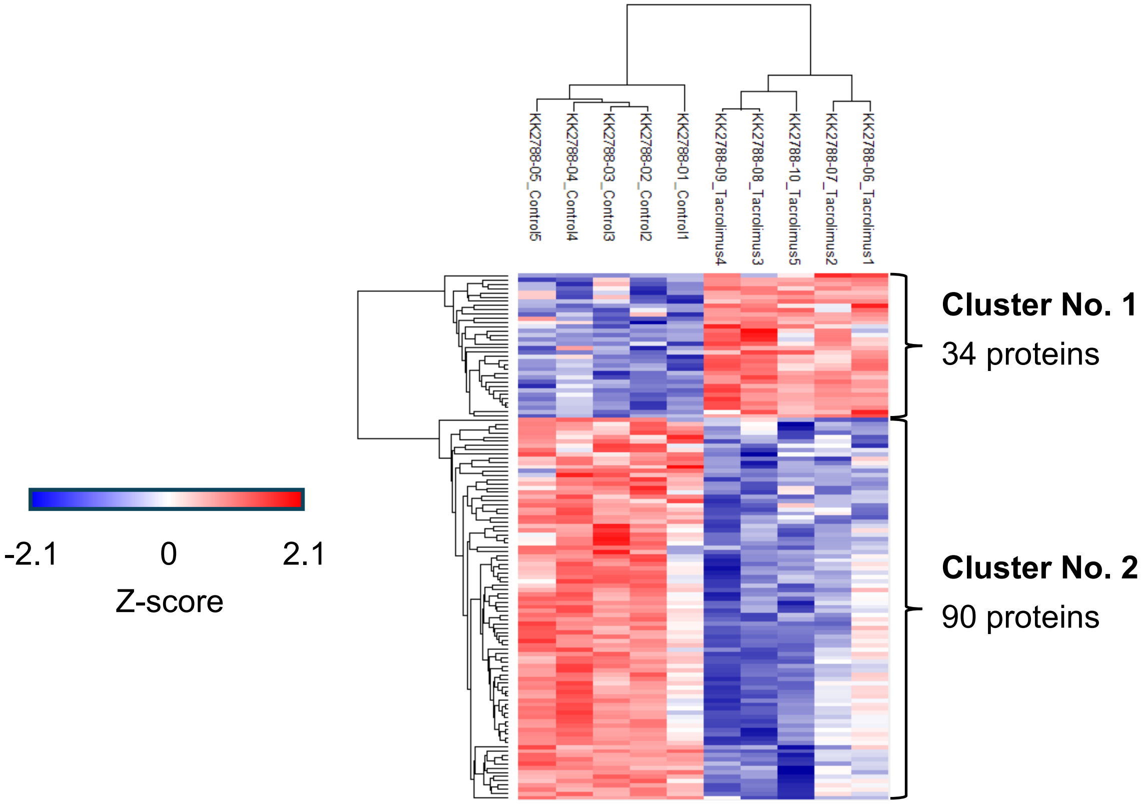

KK2788-05_Control5
KK2788-04_Control4
KK2788-03_Control3
KK2788-02_Control2
KK2788-01_Control1
KK2788-09_Tacrolimus4
KK2788-08_Tacrolimus3
KK2788-10_Tacrolimus5
KK2788-07_Tacrolimus2
KK2788-06_Tacrolimus1
Cluster No. 1
34 proteins
Cluster No. 2
90 proteins
-2.1
0
2.1
Z-score

Figure S3

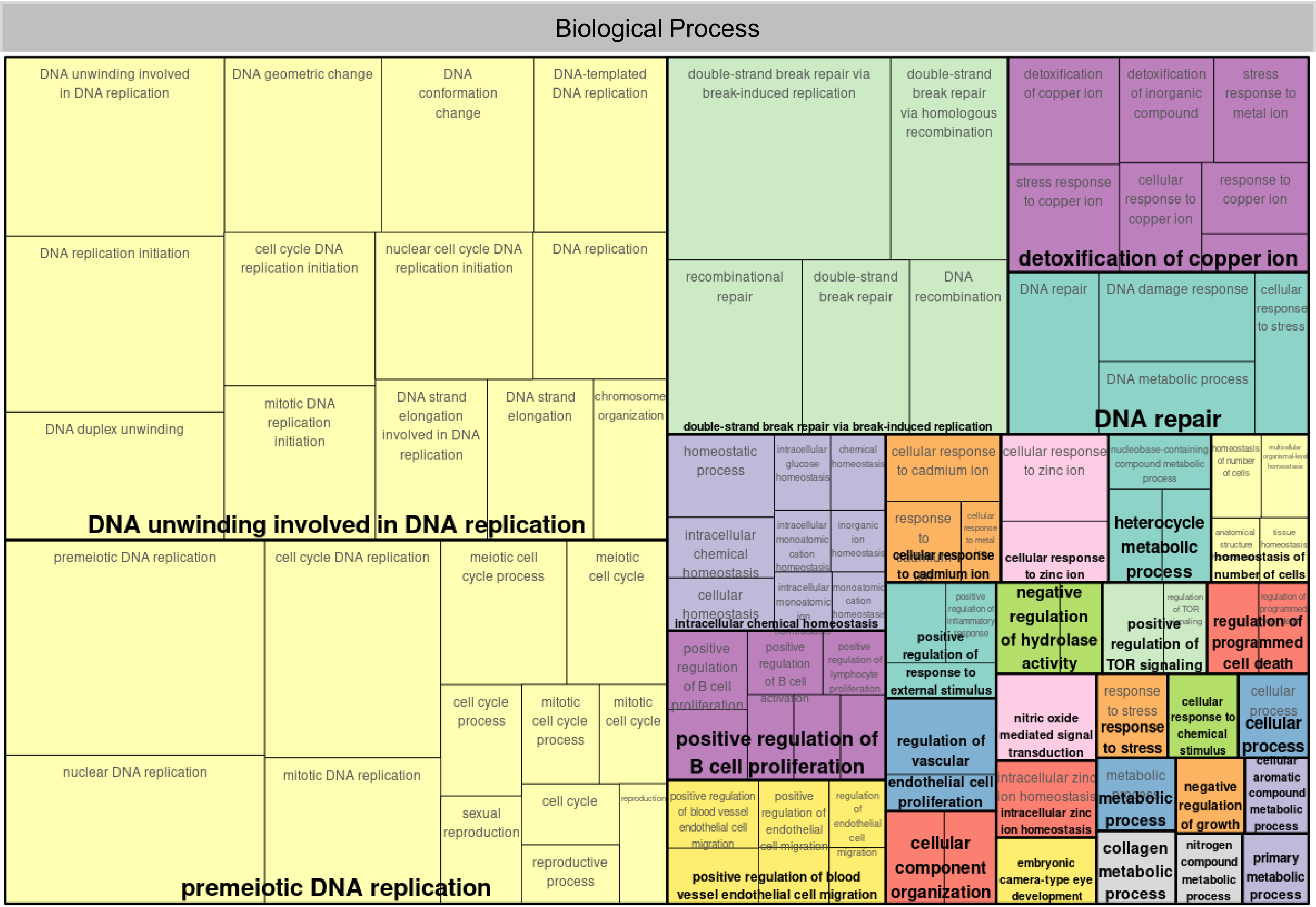
Biological Process
DNA unwinding involved in DNA replication
DNA geometric change
DNA conformation change
DNA-templated DNA replication
DNA replication initiation
cell cycle DNA replication initiation
nuclear cell cycle DNA replication initiation
DNA replication
DNA duplex unwinding
mitotic DNA replication initiation
DNA strand elongation involved in DNA replication
DNA strand elongation
chromosome organization
DNA unwinding involved in DNA replication
premeiotic DNA replication
cell cycle DNA replication
meiotic cell cycle process
meiotic cell cycle
cell cycle process
mitotic cell cycle process
mitotic cell cycle
nuclear DNA replication
mitotic DNA replication
sexual reproduction
cell cycle
reproduction
reproductive process
premeiotic DNA replication
double-strand break repair via break-induced replication
double-strand break repair via homologous recombination
recombinational repair
double-strand break repair
DNA recombination
double-strand break repair via break-induced replication
detoxification of copper ion
detoxification of inorganic compound
stress response to metal ion
stress response to copper ion
cellular response to copper ion
response to copper ion
detoxification of copper ion
DNA repair
DNA damage response
cellular response to stress
DNA metabolic process
DNA repair
homeostatic process
intracellular glucose homeostasis
chemical homeostasis
intracellular chemical homeostasis
intracellular monoatomic cation homeostasis
inorganic ion homeostasis
cellular homeostasis
intracellular monoatomic ion homeostasis
monoatomic cation homeostasis
intracellular chemical homeostasis
cellular response to cadmium ion
response to cadmium ion
cellular response to metal ion
cellular response to cadmium ion
cellular response to zinc ion
cellular response to zinc ion
nucleobase-containing compound metabolic process
heterocycle metabolic process
homeostasis of number of cells
multicellular organismal-level homeostasis
anatomical structure homeostasis
tissue homeostasis
homeostasis of number of cells
positive regulation of B cell proliferation
positive regulation of B cell activation
positive regulation of lymphocyte proliferation
positive regulation of B cell proliferation
positive regulation of inflammatory response
positive regulation of response to external stimulus
negative regulation of hydrolase activity
regulation of TOR signaling
positive regulation of TOR signaling
regulation of programmed cell death
regulation of programmed cell death
regulation of vascular endothelial cell proliferation
nitric oxide mediated signal transduction
response to stress
response to stress
cellular response to chemical stimulus
cellular process
cellular process
intracellular zinc ion homeostasis
intracellular zinc ion homeostasis
metabolic process
metabolic process
negative regulation of growth
cellular aromatic compound metabolic process
positive regulation of blood vessel endothelial cell migration
positive regulation of endothelial cell migration
regulation of endothelial cell migration
positive regulation of blood vessel endothelial cell migration
cellular component organization
embryonic camera-type eye development
collagen metabolic process
nitrogen compound metabolic process
primary metabolic process

Figure S4

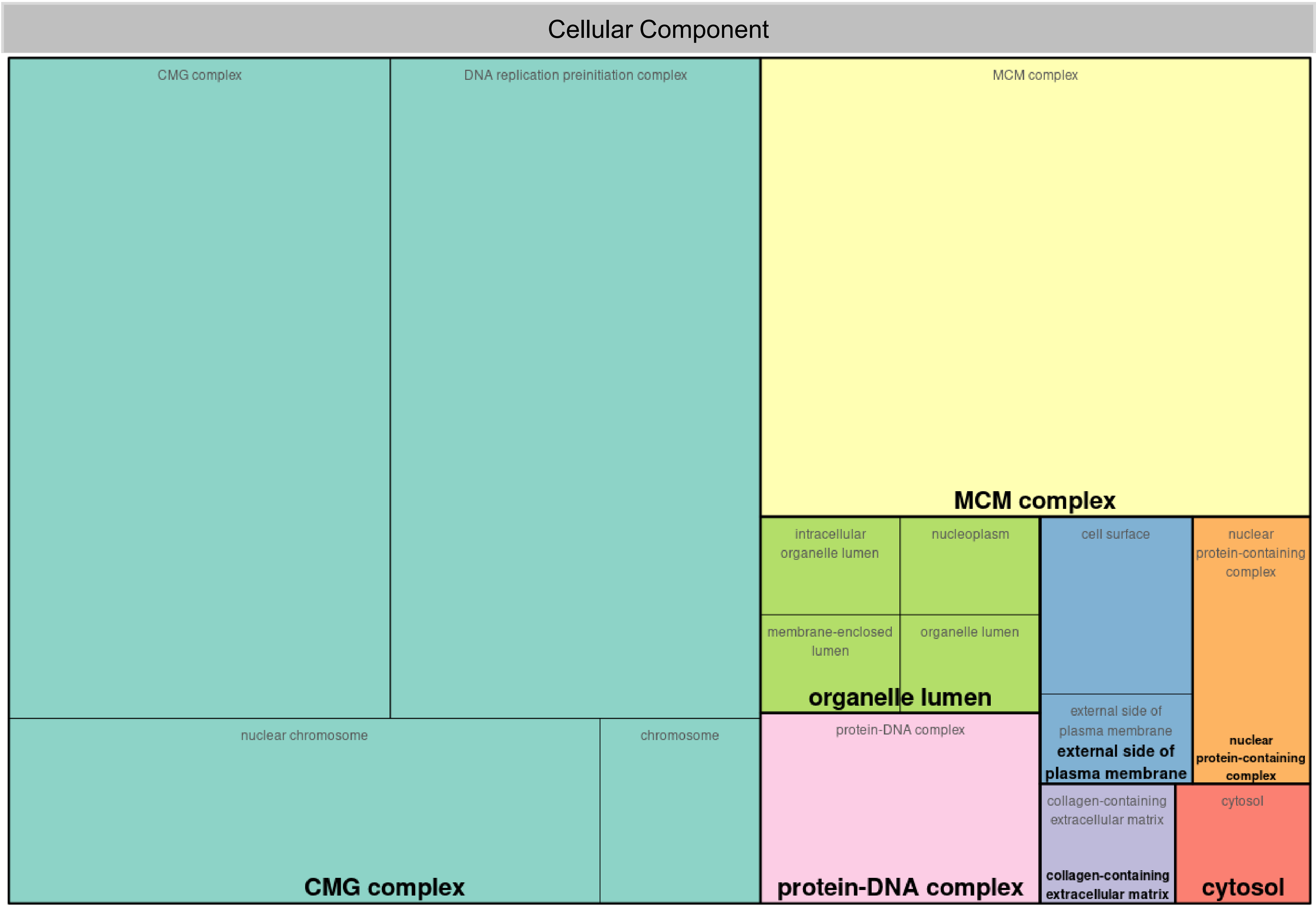

Cellular Component
CMG complex
DNA replication preinitiation complex
MCM complex
MCM complex
intracellular organelle lumen
nucleoplasm
membrane-enclosed lumen
organelle lumen
organelle lumen
cell surface
external side of plasma membrane
external side of plasma membrane
nuclear protein-containing complex
nuclear protein-containing complex
nuclear chromosome
chromosome
CMG complex
protein-DNA complex
protein-DNA complex
collagen-containing extracellular matrix
collagen-containing extracellular matrix
cytosol
cytosol

Figure S5

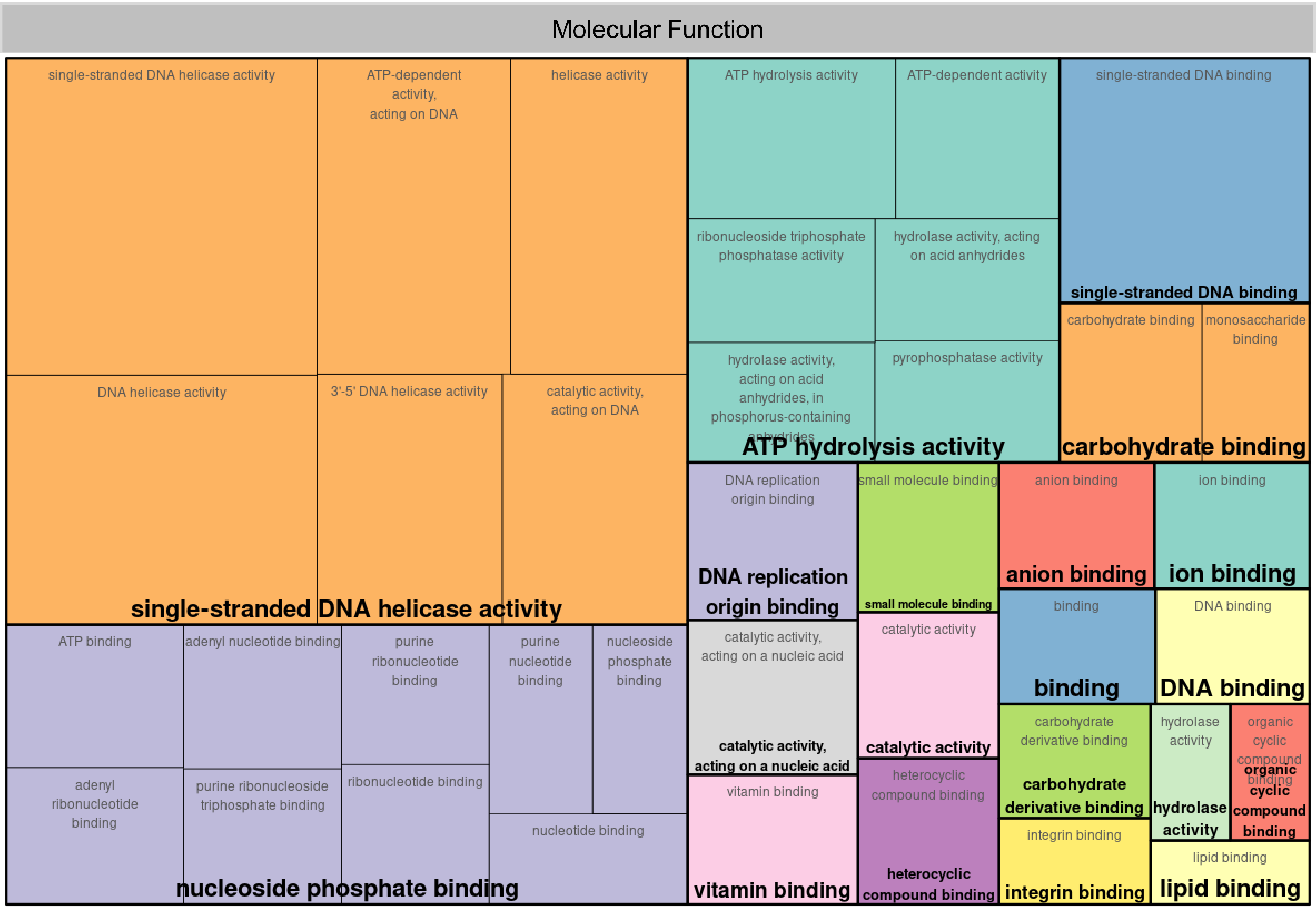

Molecular Function
single-stranded DNA helicase activity
ATP-dependent activity, acting on DNA
helicase activity
DNA helicase activity
3'-5' DNA helicase activity
catalytic activity, acting on DNA
single-stranded DNA helicase activity
ATP hydrolysis activity
ATP-dependent activity
ribonucleoside triphosphate phosphatase activity
hydrolase activity, acting on acid anhydrides
hydrolase activity, acting on acid anhydrides, in phosphorus-containing anhydrides
pyrophosphatase activity
ATP hydrolysis activity
single-stranded DNA binding
single-stranded DNA binding
carbohydrate binding
monosaccharide binding
carbohydrate binding
DNA replication origin binding
DNA replication origin binding
small molecule binding
small molecule binding
anion binding
anion binding
ion binding
ion binding
binding
binding
DNA binding
DNA binding
ATP binding
adenyl nucleotide binding
purine ribonucleotide binding
purine nucleotide binding
nucleoside phosphate binding
adenyl ribonucleotide binding
purine ribonucleoside triphosphate binding
ribonucleotide binding
nucleotide binding
nucleoside phosphate binding
catalytic activity, acting on a nucleic acid
catalytic activity, acting on a nucleic acid
catalytic activity
catalytic activity
vitamin binding
vitamin binding
heterocyclic compound binding
heterocyclic compound binding
carbohydrate derivative binding
carbohydrate derivative binding
hydrolase activity
hydrolase activity
organic cyclic compound binding
organic cyclic compound binding
integrin binding
integrin binding
lipid binding
lipid binding

Figure S6

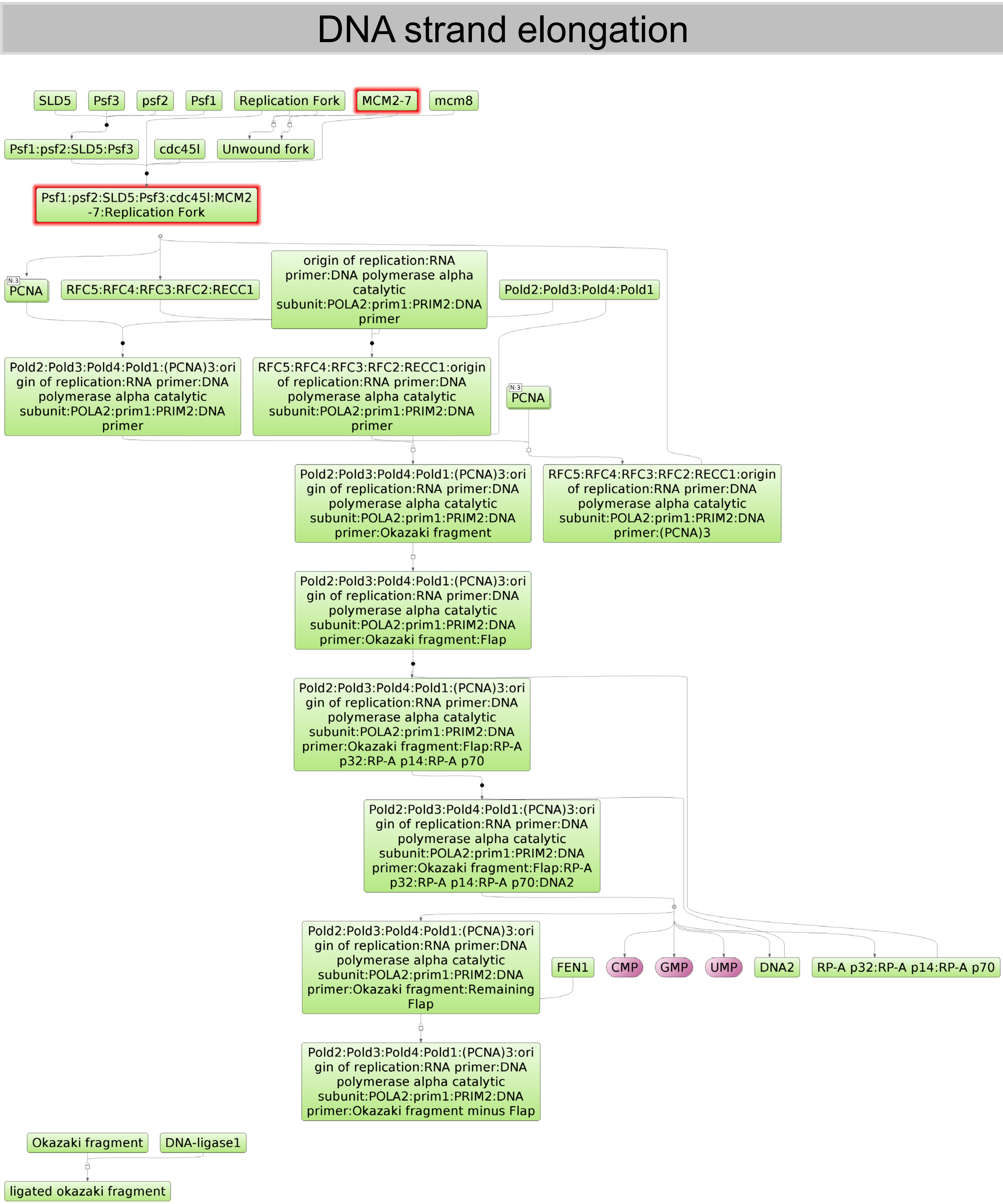
DNA strand elongation
SLD5
Psf3
psf2
Psf1
Replication Fork
MCM2-7
mcm8
Psf1:psf2:SLD5:Psf3
cdc45l
Unwound fork
Psf1:psf2:SLD5:Psf3:cdc45l:MCM2-7:Replication Fork
N:3
PCNA
RFC5:RFC4:RFC3:RFC2:RECC1
origin of replication:RNA primer:DNA polymerase alpha catalytic subunit:POLA2:prim1:PRIM2:DNA primer
Pold2:Pold3:Pold4:Pold1
Pold2:Pold3:Pold4:Pold1:(PCNA)3:origin of replication:RNA primer:DNA polymerase alpha catalytic subunit:POLA2:prim1:PRIM2:DNA primer
RFC5:RFC4:RFC3:RFC2:RECC1:origin of replication:RNA primer:DNA polymerase alpha catalytic subunit:POLA2:prim1:PRIM2:DNA primer
N:3
PCNA
Pold2:Pold3:Pold4:Pold1:(PCNA)3:origin of replication:RNA primer:DNA polymerase alpha catalytic subunit:POLA2:prim1:PRIM2:DNA primer:Okazaki fragment
RFC5:RFC4:RFC3:RFC2:RECC1:origin of replication:RNA primer:DNA polymerase alpha catalytic subunit:POLA2:prim1:PRIM2:DNA primer:(PCNA)3
Pold2:Pold3:Pold4:Pold1:(PCNA)3:origin of replication:RNA primer:DNA polymerase alpha catalytic subunit:POLA2:prim1:PRIM2:DNA primer:Okazaki fragment:Flap
Pold2:Pold3:Pold4:Pold1:(PCNA)3:origin of replication:RNA primer:DNA polymerase alpha catalytic subunit:POLA2:prim1:PRIM2:DNA primer:Okazaki fragment:Flap:RP-A p32:RP-A p14:RP-A p70
Pold2:Pold3:Pold4:Pold1:(PCNA)3:origin of replication:RNA primer:DNA polymerase alpha catalytic subunit:POLA2:prim1:PRIM2:DNA primer:Okazaki fragment:Flap:RP-A p32:RP-A p14:RP-A p70:DNA2
Pold2:Pold3:Pold4:Pold1:(PCNA)3:origin of replication:RNA primer:DNA polymerase alpha catalytic subunit:POLA2:prim1:PRIM2:DNA primer:Okazaki fragment:Remaining Flap
FEN1
CMP
GMP
UMP
DNA2
RP-A p32:RP-A p14:RP-A p70
Pold2:Pold3:Pold4:Pold1:(PCNA)3:origin of replication:RNA primer:DNA polymerase alpha catalytic subunit:POLA2:prim1:PRIM2:DNA primer:Okazaki fragment minus Flap
Okazaki fragment
DNA-ligase1
ligated okazaki fragment

Figure S7

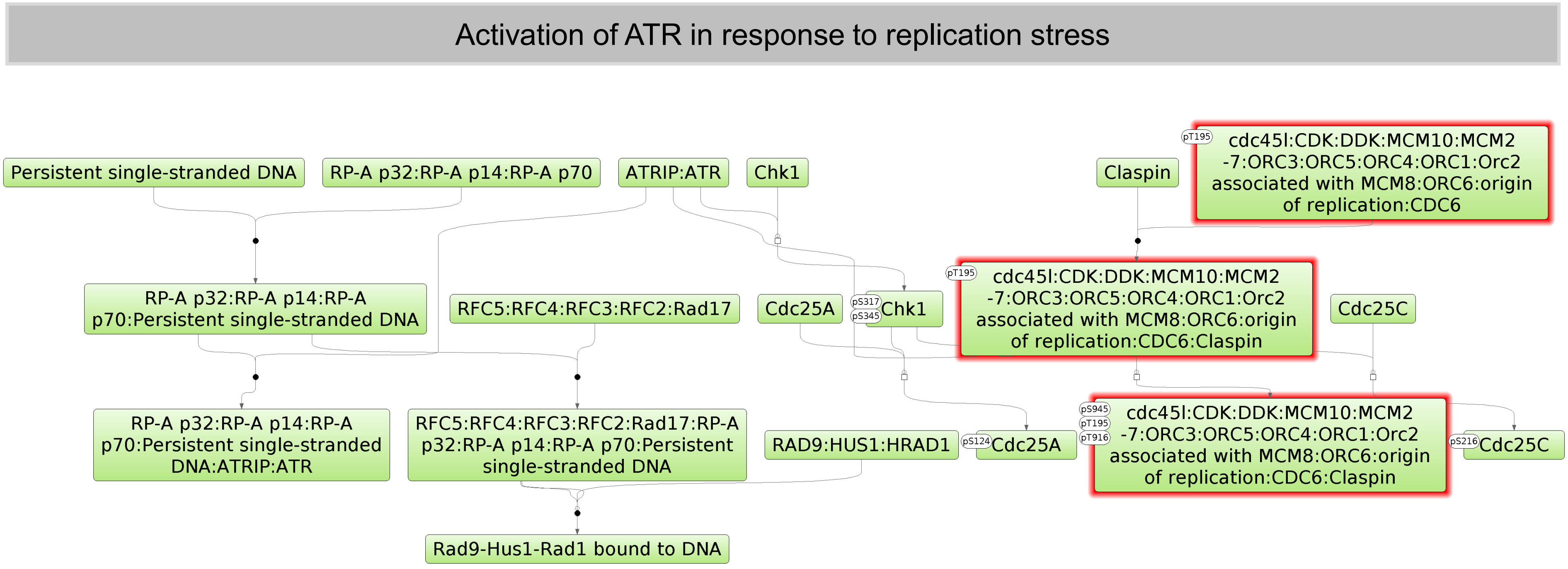

Activation of ATR in response to replication stress
Persistent single-stranded DNA
RP-A p32:RP-A p14:RP-A p70
ATRIP:ATR
Chk1
Claspin
pT195
cdc45l:CDK:DDK:MCM10:MCM2 -7:ORC3:ORC5:ORC4:ORC1:Orc2 associated with MCM8:ORC6:origin of replication:CDC6
RP-A p32:RP-A p14:RP-A p70:Persistent single-stranded DNA
RFC5:RFC4:RFC3:RFC2:Rad17
Cdc25A
pS317
pS345
Chk1
pT195
cdc45l:CDK:DDK:MCM10:MCM2 -7:ORC3:ORC5:ORC4:ORC1:Orc2 associated with MCM8:ORC6:origin of replication:CDC6:Claspin
Cdc25C
RP-A p32:RP-A p14:RP-A p70:Persistent single-stranded DNA:ATRIP:ATR
RFC5:RFC4:RFC3:RFC2:Rad17:RP-A p32:RP-A p14:RP-A p70:Persistent single-stranded DNA
RAD9:HUS1:HRAD1
pS124
Cdc25A
pS945
pT195
pT916
cdc45l:CDK:DDK:MCM10:MCM2 -7:ORC3:ORC5:ORC4:ORC1:Orc2 associated with MCM8:ORC6:origin of replication:CDC6:Claspin
pS216
Cdc25C
Rad9-Hus1-Rad1 bound to DNA

Figure S8

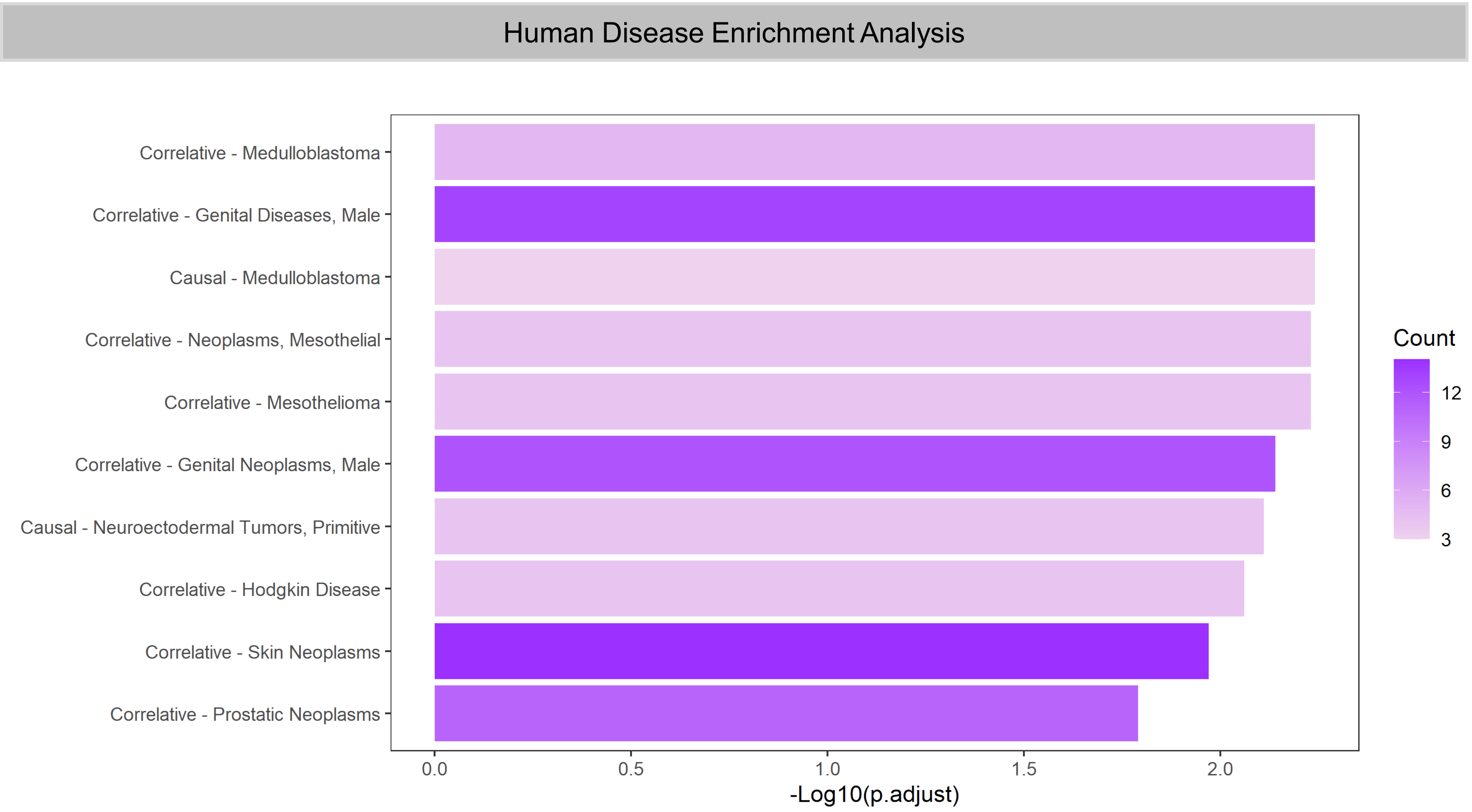
Human Disease Enrichment Analysis
Correlative - Medulloblastoma
Correlative - Genital Diseases, Male
Causal - Medulloblastoma
Correlative - Neoplasms, Mesothelial
Correlative - Mesothelioma
Correlative - Genital Neoplasms, Male
Causal - Neuroectodermal Tumors, Primitive
Correlative - Hodgkin Disease
Correlative - Skin Neoplasms
Correlative - Prostatic Neoplasms
0.0
0.5
1.0
1.5
2.0
-Log10(p.adjust)
Count
12
9
6
3

Figure S9

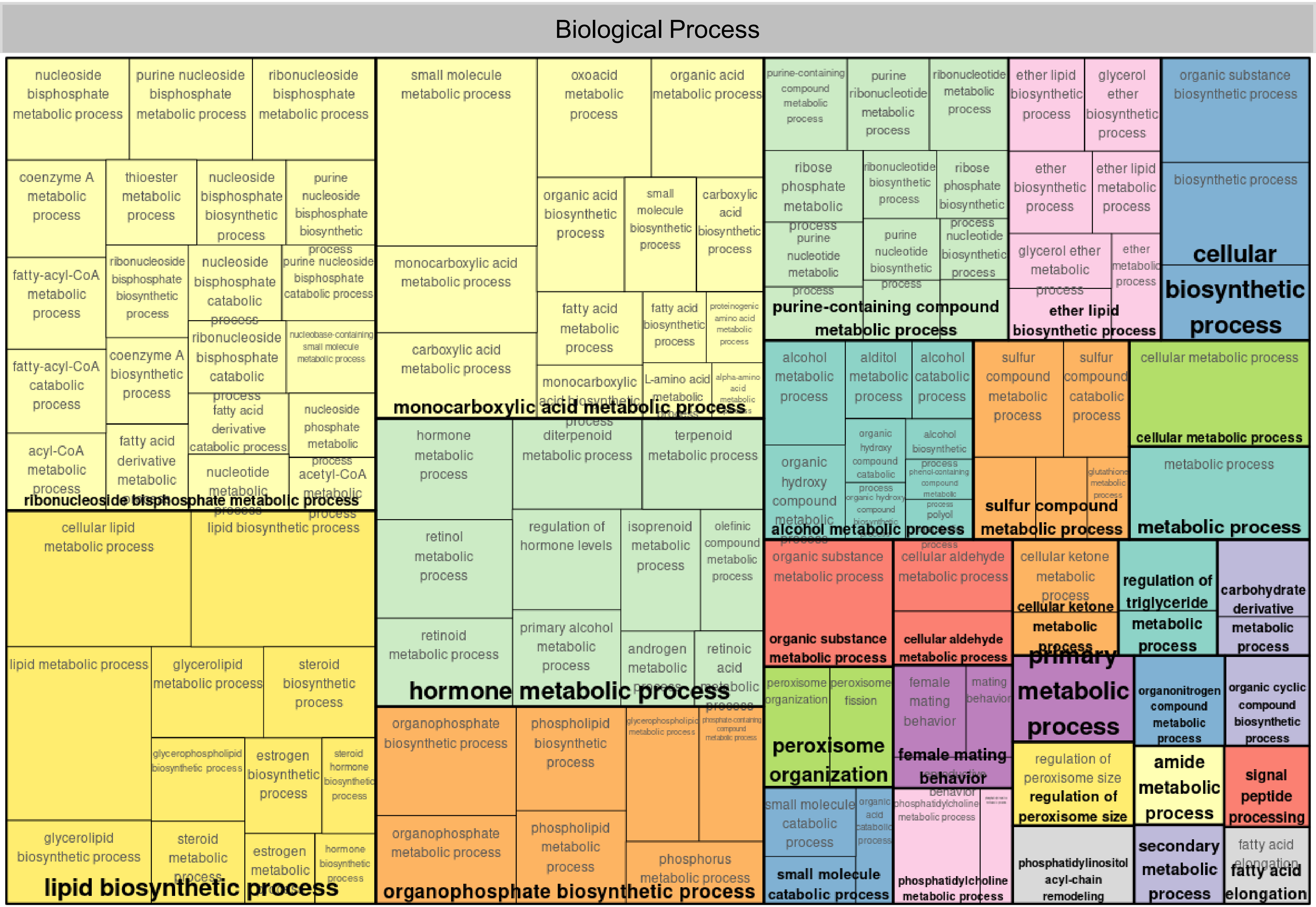

Biological Process
ribonucleoside bisphosphate metabolic process
nucleoside bisphosphate metabolic process
purine nucleoside bisphosphate metabolic process
ribonucleoside bisphosphate metabolic process
coenzyme A metabolic process
thioester metabolic process
nucleoside bisphosphate biosynthetic process
purine nucleoside bisphosphate biosynthetic process
fatty-acyl-CoA metabolic process
ribonucleoside bisphosphate biosynthetic process
nucleoside bisphosphate catabolic process
purine nucleoside bisphosphate catabolic process
fatty-acyl-CoA catabolic process
coenzyme A biosynthetic process
ribonucleoside bisphosphate catabolic process
nucleobase-containing small molecule metabolic process
fatty acid derivative catabolic process
nucleoside phosphate metabolic process
acyl-CoA metabolic process
fatty acid derivative metabolic process
nucleotide metabolic process
acetyl-CoA metabolic process
monocarboxylic acid metabolic process
small molecule metabolic process
oxoacid metabolic process
organic acid metabolic process
organic acid biosynthetic process
small molecule biosynthetic process
carboxylic acid biosynthetic process
monocarboxylic acid metabolic process
fatty acid metabolic process
fatty acid biosynthetic process
proteinogenic amino acid metabolic process
carboxylic acid metabolic process
monocarboxylic acid biosynthetic process
L-amino acid metabolic process
alpha-amino acid metabolic process
purine-containing compound metabolic process
purine-containing compound metabolic process
purine ribonucleotide metabolic process
ribonucleotide metabolic process
ribose phosphate metabolic process
ribonucleotide biosynthetic process
ribose phosphate biosynthetic process
purine nucleotide metabolic process
purine nucleotide biosynthetic process
nucleotide biosynthetic process
ether lipid biosynthetic process
ether lipid biosynthetic process
glycerol ether biosynthetic process
ether biosynthetic process
ether lipid metabolic process
glycerol ether metabolic process
ether metabolic process
cellular biosynthetic process
organic substance biosynthetic process
biosynthetic process
lipid biosynthetic process
cellular lipid metabolic process
lipid biosynthetic process
lipid metabolic process
glycerolipid metabolic process
steroid biosynthetic process
glycerophospholipid biosynthetic process
estrogen biosynthetic process
steroid hormone biosynthetic process
glycerolipid biosynthetic process
steroid metabolic process
estrogen metabolic process
hormone biosynthetic process
hormone metabolic process
hormone metabolic process
diterpenoid metabolic process
terpenoid metabolic process
retinol metabolic process
regulation of hormone levels
isoprenoid metabolic process
olefinic compound metabolic process
retinoid metabolic process
primary alcohol metabolic process
androgen metabolic process
retinoic acid metabolic process
organophosphate biosynthetic process
organophosphate biosynthetic process
phospholipid biosynthetic process
glycerophospholipid metabolic process
phosphate-containing compound metabolic process
organophosphate metabolic process
phospholipid metabolic process
phosphorus metabolic process
alcohol metabolic process
alcohol metabolic process
alditol metabolic process
alcohol catabolic process
organic hydroxy compound catabolic process
alcohol biosynthetic process
phenol-containing compound metabolic process
organic hydroxy compound metabolic process
organic hydroxy compound biosynthetic process
polyol
sulfur compound metabolic process
sulfur compound metabolic process
sulfur compound catabolic process
glutathione metabolic process
metabolic process
cellular metabolic process
cellular metabolic process
metabolic process
organic substance metabolic process
organic substance metabolic process
cellular aldehyde metabolic process
cellular aldehyde metabolic process
cellular ketone metabolic process
cellular ketone metabolic process
regulation of triglyceride metabolic process
carbohydrate derivative metabolic process
primary metabolic process
peroxisome organization
peroxisome organization
peroxisome fission
female mating behavior
female mating behavior
mating behavior
organonitrogen compound metabolic process
organic cyclic compound biosynthetic process
regulation of peroxisome size
regulation of peroxisome size
amide metabolic process
signal peptide processing
small molecule catabolic process
small molecule catabolic process
organic acid catabolic process
phosphatidylcholine metabolic process
phosphatidylcholine metabolic process
phosphatidylinositol acyl-chain remodeling
secondary metabolic process
fatty acid elongation
fatty acid elongation

Figure S10

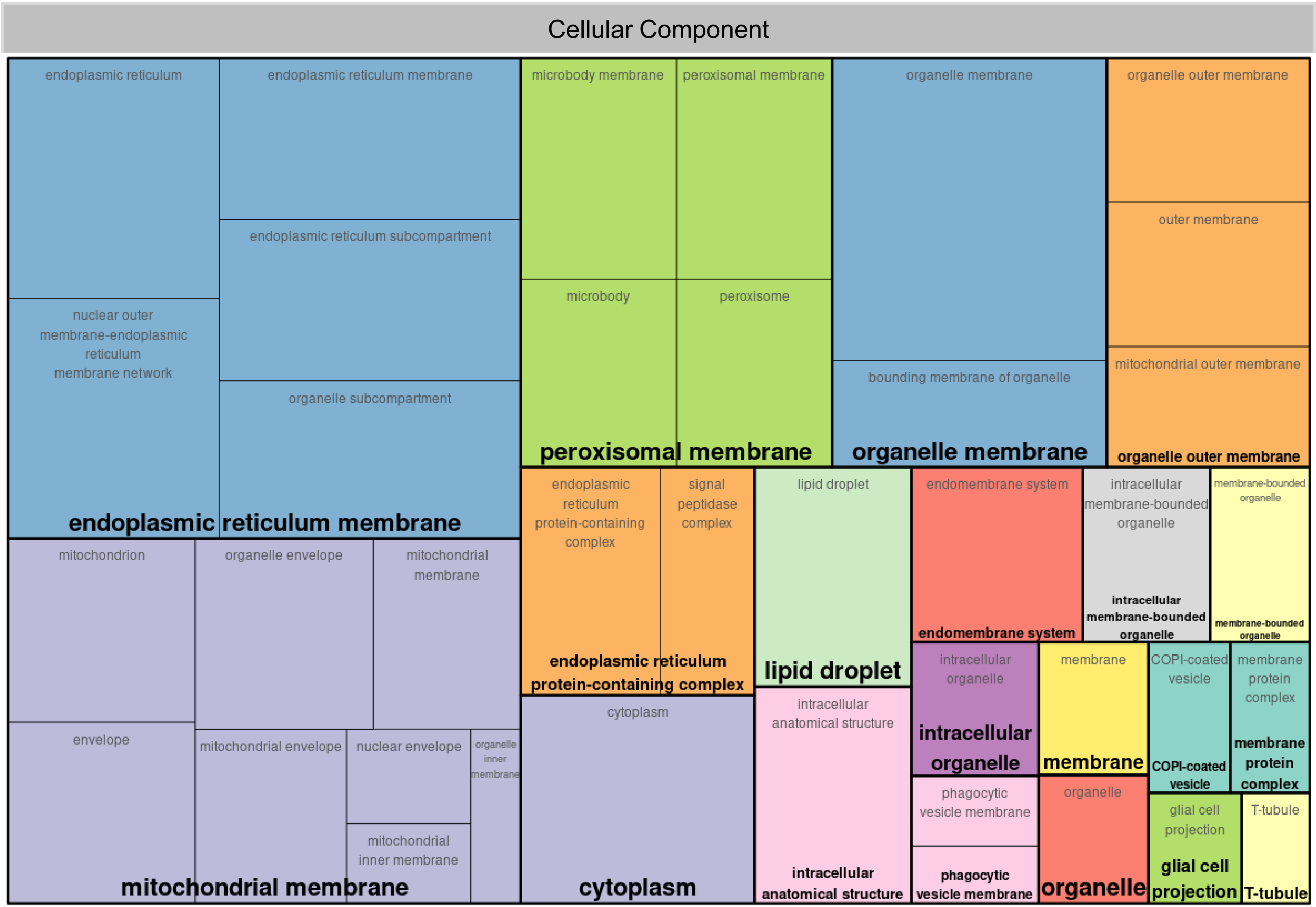

Cellular Component
endoplasmic reticulum
nuclear outer membrane-endoplasmic reticulum membrane network
endoplasmic reticulum membrane
endoplasmic reticulum subcompartment
organelle subcompartment
endoplasmic reticulum membrane
microbody membrane
peroxisomal membrane
microbody
peroxisome
peroxisomal membrane
organelle membrane
bounding membrane of organelle
organelle membrane
organelle outer membrane
outer membrane
mitochondrial outer membrane
organelle outer membrane
mitochondrion
envelope
organelle envelope
mitochondrial membrane
mitochondrial envelope
nuclear envelope
organelle inner membrane
mitochondrial inner membrane
mitochondrial membrane
endoplasmic reticulum protein-containing complex
signal peptidase complex
endoplasmic reticulum protein-containing complex
cytoplasm
cytoplasm
lipid droplet
lipid droplet
intracellular anatomical structure
intracellular anatomical structure
endomembrane system
endomembrane system
intracellular membrane-bounded organelle
intracellular membrane-bounded organelle
membrane-bounded organelle
membrane-bounded organelle
intracellular organelle
intracellular organelle
phagocytic vesicle membrane
phagocytic vesicle membrane
membrane
membrane
organelle
organelle
COPI-coated vesicle
COPI-coated vesicle
membrane protein complex
membrane protein complex
glial cell projection
glial cell projection
T-tubule
T-tubule

Figure S11

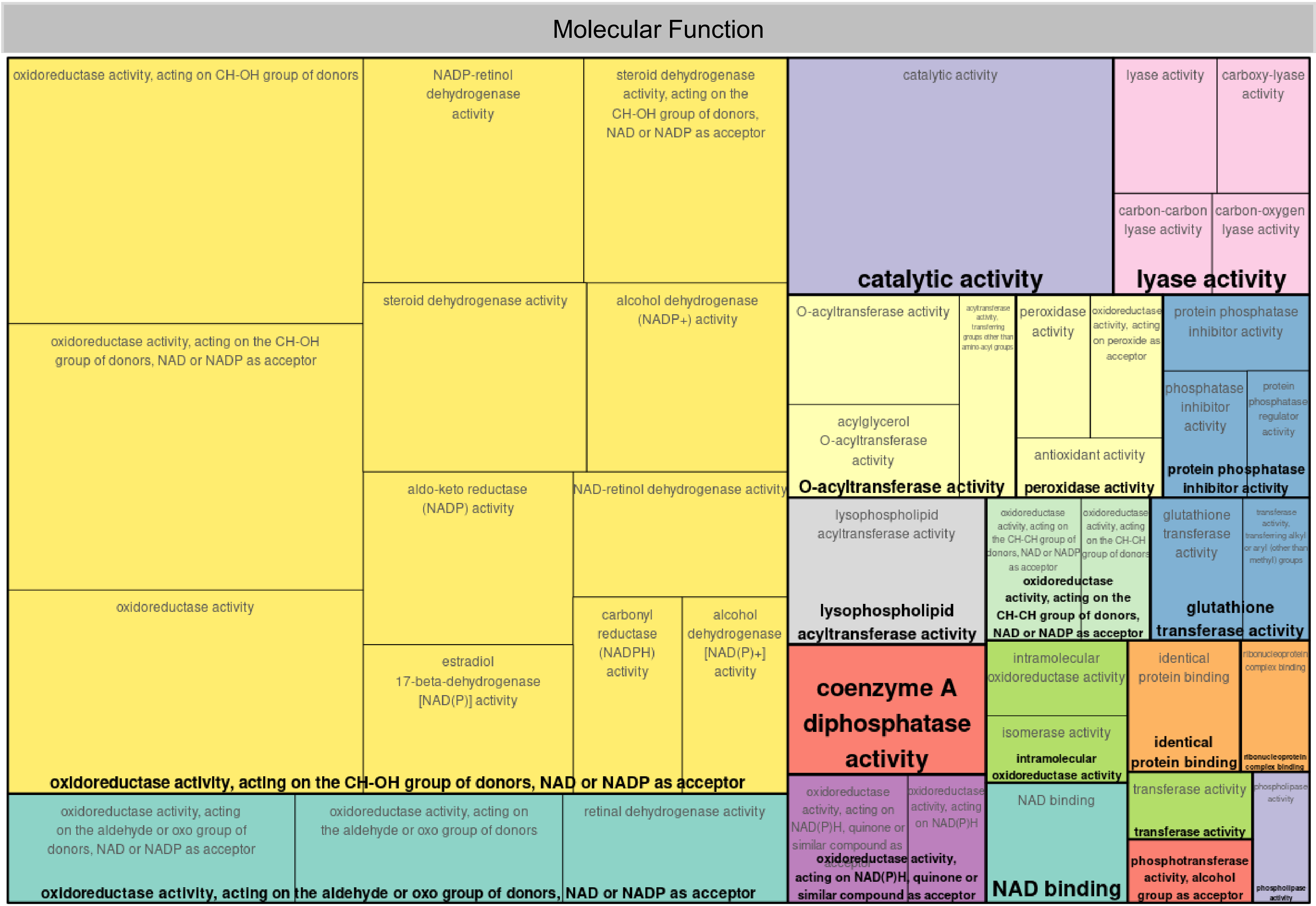

Molecular Function
oxidoreductase activity, acting on CH-OH group of donors
NADP-retinol dehydrogenase activity
steroid dehydrogenase activity, acting on the CH-OH group of donors, NAD or NADP as acceptor
oxidoreductase activity, acting on the CH-OH group of donors, NAD or NADP as acceptor
steroid dehydrogenase activity
alcohol dehydrogenase (NADP+) activity
oxidoreductase activity
aldo-keto reductase (NADP) activity
NAD-retinol dehydrogenase activity
estradiol 17-beta-dehydrogenase [NAD(P)] activity
carbonyl reductase (NADPH) activity
alcohol dehydrogenase [NAD(P)+] activity
oxidoreductase activity, acting on the CH-OH group of donors, NAD or NADP as acceptor
oxidoreductase activity, acting on the aldehyde or oxo group of donors, NAD or NADP as acceptor
oxidoreductase activity, acting on the aldehyde or oxo group of donors
retinal dehydrogenase activity
oxidoreductase activity, acting on the aldehyde or oxo group of donors, NAD or NADP as acceptor
catalytic activity
catalytic activity
lyase activity
carboxy-lyase activity
carbon-carbon lyase activity
carbon-oxygen lyase activity
lyase activity
O-acyltransferase activity
acyltransferase activity, transferring groups other than amino-acyl groups
acylglycerol O-acyltransferase activity
O-acyltransferase activity
peroxidase activity
oxidoreductase activity, acting on peroxide as acceptor
antioxidant activity
peroxidase activity
protein phosphatase inhibitor activity
phosphatase inhibitor activity
protein phosphatase regulator activity
protein phosphatase inhibitor activity
lysophospholipid acyltransferase activity
lysophospholipid acyltransferase activity
oxidoreductase activity, acting on the CH-CH group of donors, NAD or NADP as acceptor
oxidoreductase activity, acting on the CH-CH group of donors
oxidoreductase activity, acting on the CH-CH group of donors, NAD or NADP as acceptor
glutathione transferase activity
transferase activity, transferring alkyl or aryl (other than methyl) groups
glutathione transferase activity
coenzyme A diphosphatase activity
intramolecular oxidoreductase activity
isomerase activity
intramolecular oxidoreductase activity
identical protein binding
ribonucleoprotein complex binding
identical protein binding
ribonucleoprotein complex binding
oxidoreductase activity, acting on NAD(P)H, quinone or similar compound as acceptor
oxidoreductase activity, acting on NAD(P)H
oxidoreductase activity, acting on NAD(P)H, quinone or similar compound as acceptor
NAD binding
NAD binding
transferase activity
transferase activity
phospholipase activity
phosphotransferase activity, alcohol group as acceptor
phospholipase activity

Figure S12

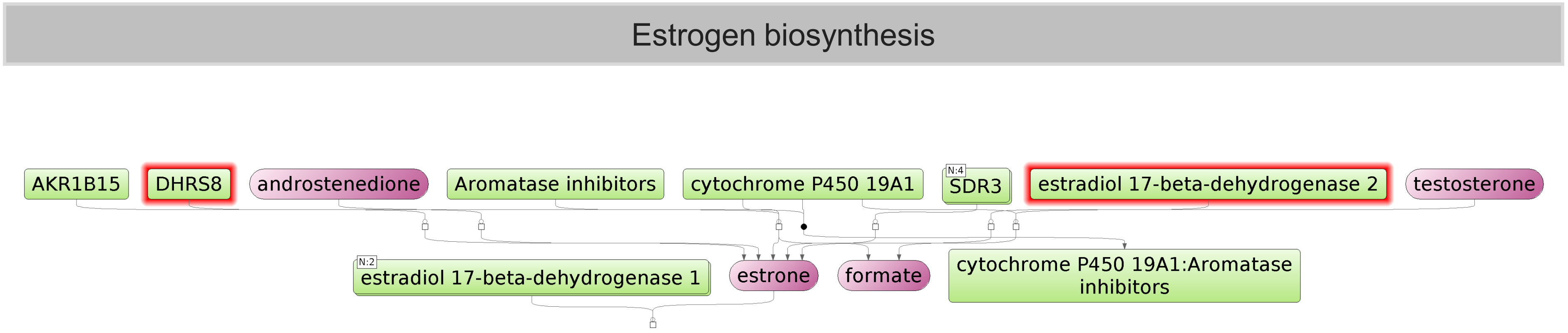
Estrogen biosynthesis
AKR1B15
DHRS8
androstenedione
Aromatase inhibitors
cytochrome P450 19A1
N:4
SDR3
estradiol 17-beta-dehydrogenase 2
testosterone
N:2
estradiol 17-beta-dehydrogenase 1
estrone
formate
cytochrome P450 19A1:Aromatase inhibitors

Figure S13

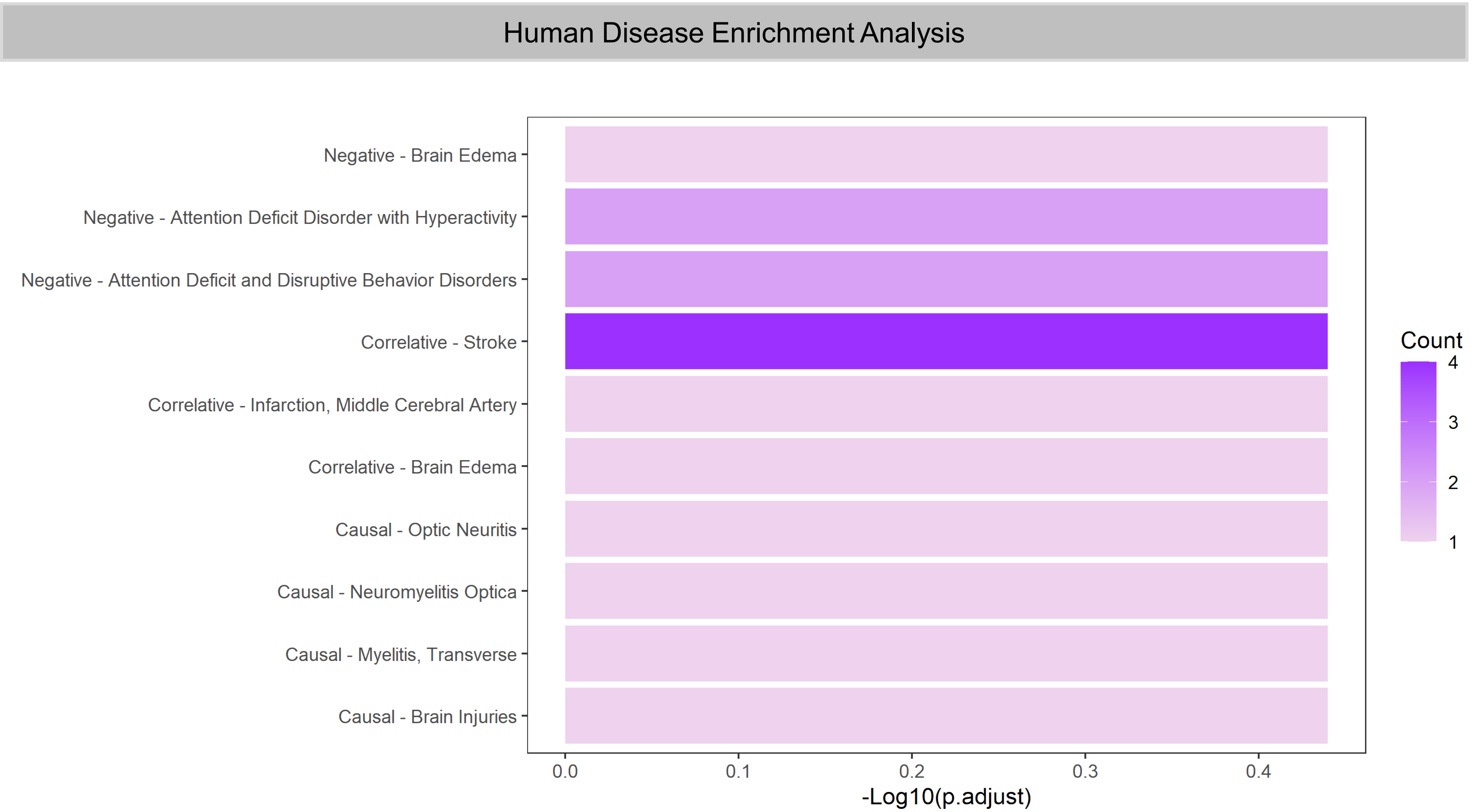
Human Disease Enrichment Analysis
Negative - Brain Edema
Negative - Attention Deficit Disorder with Hyperactivity
Negative - Attention Deficit and Disruptive Behavior Disorders
Correlative - Stroke
Correlative - Infarction, Middle Cerebral Artery
Correlative - Brain Edema
Causal - Optic Neuritis
Causal - Neuromyelitis Optica
Causal - Myelitis, Transverse
Causal - Brain Injuries
0.0
0.1
0.2
0.3
0.4
-Log10(p.adjust)
Count
4
3
2
1

Supplementary Figure Legends

Figure S1. Differentially expressed proteins identified based on statistical significance and $\log_2$ fold change thresholds. (a) Volcano plot showing proteins with significant changes ($P < 0.05$, t-test) after $\log_2$ transformation and missing value imputation. The y-axis represents $-\log_{10}$ (*p*-value), and the x-axis represents $\log_2$ (TAC/Control). The red dotted line indicates a $\log_2$ fold change of +1, the blue dotted line indicates a $\log_2$ fold change of −1. The horizontal gray dotted line represents $p = 0.05$. Red dots indicate upregulated proteins, blue dots indicate downregulated proteins, and gray dots indicate proteins without significant change. (b) After $\log_2$ transformation and missing value imputation, a two-group t-test was performed. Proteins showing significant differences ($p< 0.05$) with a $\log_2$ fold change ≥ 1 or ≤ −1 were extracted, and their quantitative values were converted to Z-scores to generate a heatmap.

Figure S2. Differentially expressed proteins identified with permutation-based FDR < 0.05. After $\log_2$ transformation and missing value imputation, a t-test was performed. To correct for multiple testing, permutation-based FDR was applied (250 permutations; FDR 0.05; $S_0 = 0.1$). (a) Volcano plot showing proteins with FDR < 0.05. The y-axis represents $-\log_{10}$(*p*-value), and the x-axis represents $\log_2$ (TAC/Control). The boundary line indicates FDR = 0.05. Red dots indicate increased proteins, blue dots indicate decreased proteins, and gray dots indicate no significant change. (b) Proteins with significant differences (FDR < 0.05) were extracted, and their quantitative values were converted to Z-scores to generate a heatmap.

Figure S3. Gene Ontology (GO) analysis of proteins upregulated in the TAC-treated group (*Biological Process* category). A tree map was generated based on adjusted *p*-values to provide a global overview of enriched biological processes.

Figure S4. Gene Ontology (GO) analysis of proteins upregulated in the TAC-treated group (*Cellular Component* category). A tree map was generated based on adjusted *p*-values to provide a macroscopic overview of enriched cellular component.

Figure S5. Gene Ontology (GO) analysis of proteins upregulated in the TAC-treated group (*Molecular Function* category). A tree map was generated based on adjusted *p*-values to provide an overview of enriched molecular functions.

Figure S6. Pathway analysis of proteins upregulated in TAC-treated samples: *DNA strand elongation*. Enrichment analysis was performed using curated pathway databases to provide an overview of biological trends associated with TAC exposure, with particular emphasis on *DNA strand elongation* as a representative upregulated pathway. In the pathway diagram, green, rounded rectangles denote macromolecules, representing individual proteins, protein complexes, or related structures. Those outlined in red correspond to molecules identified as significantly associated based on the enrichment analysis. A small element positioned at the upper left of a green, rounded rectangle indicates additional molecular information; for example, an element labeled "N = 3" specifies that the macromolecule forms a trimer. Pink capsule-shaped elements represent simple chemicals included in the pathway. Regarding edges, a black arrow indicates production, an arrowhead accompanied by a white circle represents catalysis, a black circle denotes a reaction (association), a double circle indicates a reaction (dissociation), and a white-outlined square represents a reaction classified as a process.

Figure S7. Pathway analysis of proteins upregulated in TAC-treated samples: *ATR activation in response to replication stress*. Enrichment analysis was performed using curated pathways, with particular emphasis on the *Activation of ATR in response to replication stress* pathway. In the pathway diagram, green, rounded rectangles denote macromolecules representing individual proteins, protein complexes, or related molecular structures. Molecules outlined in red indicate those identified as significantly associated based on the enrichment analysis. A small capsule-shaped element positioned at the upper left of a green, rounded rectangle indicates additional molecular information. In this figure, labels such as "pT195," "pS945," "pT916," "pS317," "pS345," "pS124," and "pS216" indicate phosphorylation at corresponding threonine or serine residues. Regarding edges, a black arrow represents production, a black circle on the edge denotes a reaction (association), and a white-outlined square indicates a reaction categorized as a process.

Figure S8. Human disease enrichment analysis of proteins upregulated in the TAC-treated group. Top 10 enriched human disease terms identified by enrichment analysis, shown as a horizontal bar graph. Bars represent the enrichment score calculated as $-\log_{10}$ (adjusted *p*-value), and disease names are listed on the y-axis. Prefixes such as "Correlative" and "Causal" in disease names reflect the annotation structure of the database. "Correlative" indicates statistical co-occurrence or association reported in the literature, whereas "Causal" denotes curated evidence suggesting a causative relationship. These labels do not imply that the corresponding biological processes are activated in kidney tissue, but rather represent literature-based associations assigned in the database.

Figure S9. Gene Ontology (GO) analysis of proteins downregulated in the TAC-treated group (*Biological Process* category). A tree map was generated based on adjusted *p*-values to provide a global overview of enriched biological processes.

Figure S10. Gene Ontology (GO) analysis of proteins downregulated in the TAC-treated group (*Cellular Component* category). A tree map was generated based on adjusted *p*-values to provide a global overview of enriched cellular components.

Figure S11. Gene Ontology (GO) analysis of proteins downregulated in the TAC-treated group (*Molecular Function* category). A tree map was generated based on adjusted *p*-values to provide a global overview of enriched molecular functions.

Figure S12. Pathway analysis of proteins downregulated in TAC-treated samples: *Estrogen biosynthesis*. Enrichment analysis was performed using pathways curated in the database, and results are presented to provide an overview of enriched pathways, with particular emphasis on the *Estrogen biosynthesis* pathway. In the pathway diagram, green, rounded rectangles denote macromolecules, representing individual proteins, protein complexes, or related molecular structures. Molecules outlined in red indicate those identified as significantly associated based on the enrichment analysis. A small capsule-shaped element positioned at the upper left of a green, rounded rectangle provides additional molecular information. In this figure, labels such as "N = 2" and "N = 4" indicate dimeric and tetrameric forms of the macromolecule, respectively. Pink capsule-shaped elements represent simple chemicals included in the pathway. Regarding edges, a black arrow indicates production, a black circle on the edge denotes a reaction (association), and a white-outlined square represents a reaction categorized as a process.

Figure S13. Human disease enrichment analysis of proteins downregulated in the TAC-treated group. The top 10 enriched human disease terms identified by enrichment analysis, shown as a horizontal bar graph. Bars represent the enrichment score calculated as $-\log_{10}$ (adjusted *p*-value), and disease names are listed on the y-axis. Prefixes such as "Negative," "Correlative," and "Causal" in the disease names reflect the annotation structure of the database. "Negative" denotes an inverse association reported in the literature, "Correlative" indicates statistical co-occurrence or association, and "Causal" represents curated evidence suggesting a causative link. These labels should be interpreted as literature-based associations assigned in the database and do not indicate activation or suppression of CNS-specific biological programs in kidney tissue.
